# CXCR3-Dependent CD4^+^ T Cells Are Required to Activate Inflammatory Monocytes for Defense against Intestinal Infection

**DOI:** 10.1371/journal.ppat.1003706

**Published:** 2013-10-10

**Authors:** Sara B. Cohen, Kirk J. Maurer, Charlotte E. Egan, Steve Oghumu, Abhay R. Satoskar, Eric Y. Denkers

**Affiliations:** 1 Department of Microbiology and Immunology, College of Veterinary Medicine, Cornell University, Ithaca, New York, United States of America; 2 Department of Biomedical Sciences, College of Veterinary Medicine, Cornell University, Ithaca, New York, United States of America; 3 Center for Animal Resources and Education, College of Veterinary Medicine, Cornell University, Ithaca, New York, United States of America; 4 Department of Pathology, The Ohio State University Medical Center, Columbus, Ohio, United States of America; Washington University School of Medicine, United States of America

## Abstract

Chemokines and their receptors play a critical role in orchestrating immunity to microbial pathogens, including the orally acquired Th1-inducing protozoan parasite *Toxoplasma gondii*. Chemokine receptor CXCR3 is associated with Th1 responses, and here we use bicistronic CXCR3-eGFP knock-in reporter mice to demonstrate upregulation of this chemokine receptor on CD4^+^ and CD8^+^ T lymphocytes during *Toxoplasma* infection. We show a critical role for CXCR3 in resistance to the parasite in the intestinal mucosa. Absence of the receptor in *Cxcr3^−/−^* mice resulted in selective loss of ability to control *T. gondii* specifically in the lamina propria compartment. CD4^+^ T cells were impaired both in their recruitment to the intestinal lamina propria and in their ability to secrete IFN-γ upon stimulation. Local recruitment of CD11b^+^Ly6C/G^+^ inflammatory monocytes, recently reported to be major anti-*Toxoplasma* effectors in the intestine, was not impacted by loss of CXCR3. However, inflammatory monocyte activation status, as measured by dual production of TNF-α and IL-12, was severely impaired in *Cxcr3^−/−^* mice. Strikingly, adoptive transfer of wild-type but not *Ifnγ^−/−^* CD4^+^ T lymphocytes into *Cxcr3^−/−^* animals prior to infection corrected the defect in inflammatory macrophage activation, simultaneously reversing the susceptibility phenotype of the knockout animals. Our results establish a central role for CXCR3 in coordinating innate and adaptive immunity, ensuring generation of Th1 effectors and their trafficking to the frontline of infection to program microbial killing by inflammatory monocytes.

## Author Summary

Inflammatory monocytes have recently emerged as important effectors in intestinal defense against enteric pathogens, but requirements for their activation are poorly defined. Here we use the protozoan *Toxoplasma gondii*, an orally acquired Th1-inducing pathogen, to study the requirements for inflammatory macrophage activation in the intestinal mucosa. We find that CD4^+^ T lymphocytes, recruited in dependence upon their expression of the chemokine receptor CXCR3, mediate activation of intestinal mucosa inflammatory monocytes via secretion of IFN-γ, in turn resulting in control of infection. Thus, CXCR3 functions as a critical lynchpin coordinating anti-microbial communication between T lymphocytes and inflammatory monocyte effectors.

## Introduction

The intestinal mucosa is a critical effector site for elimination of enteric pathogens. *Toxoplasma gondii*, a ubiquitous protozoan parasite, is a prime example of such a pathogen. Mammals are infected with *T. gondii* primarily by the ingestion of tissue cysts from undercooked meat or oocysts excreted in the feces of felines, which are the sole definitive hosts. Upon infection, the parasite induces a potent Th1 immune response that is characterized by high levels of IL-12 and IFN-γ [1], [2]. Initial IL-12 production is largely the result of MyD88-dependent Toll-like receptor (TLR) signaling in dendritic cells, and the parasite profilin molecule has been identified as a ligand for TLR11 and TLR12 [3]–[7]. IL-12 activates natural killer (NK) cells to initiate IFN-γ production and promotes T-cell differentiation towards a Th1 program. Ultimately IFN-γ is the critical cytokine involved in controlling *Toxoplasma*. While *in vitro* experiments suggest that macrophages activated by this cytokine acquire anti-*Toxoplasma* activity through upregulation of immunity-related GTPase (IRG) molecules that mediate destruction of the parasitophorous vacuole [8]–[10], the *in vivo* function of IFN-γ is less clear.

Inflammatory monocytes are an important component of defense against microbial pathogens, including *Toxoplasma* [11]. These cells express high levels of Ly6C/G (Gr-1) and are recruited from the bone marrow via chemokine (C-C motif) receptor 2 (CCR2) [12]. During *Listeria monocytogenes* infection, inflammatory monocytes are recruited from the bone marrow to the spleen and liver where they differentiate into TNF-α- and nitric oxide (NO)-producing DCs (Tip-DCs). There they are essential for bacterial clearance and mouse survival [13], [14]. Likewise, CCR2-dependent inflammatory monocytes are recruited to the lung during *Mycobacteria tuberculosis* infection where they protect mice from disease by recruiting and activating T cells and by producing NO [15], [16]. Mucosal defense against *T. gondii* has also recently been shown to require CCR2-dependent inflammatory monocytes [11]. Upon recruitment to the small intestine, these cells control the parasite either indirectly by production of IL-12 and TNF-α or directly through production of NO and IRG proteins [4]–[6], [8], [9], [11], [17]. While CCR2 enables recruitment of inflammatory monocytes to sites of infection, the factors that coordinate their activation and acquisition of effector function are not known.

CXCR3 is a Th1-associated chemokine receptor, and cells expressing this receptor respond to the IFN-γ-inducible chemokines CXCL9, 10, and 11 [18]. The receptor is expressed predominantly by T cells and NK cells and is rapidly upregulated upon cell activation. There is evidence that CXCR3 expression enables T-cell entry into sites of infection, although the outcome of recruitment varies among pathogens. In the case of *Leishmania major*, recruitment is protective as CXCR3-expressing T cells are required for the resolution of cutaneous lesions [19]. However, in the case of *Plasmodium berghei* ANKA, CXCR3 is pathogenic because it allows entry of proinflammatory cells into the CNS, resulting in cerebral malaria [20].

Here we determined the role of CXCR3 in the intestinal immune response to *Toxoplasma*. We found that loss of CXCR3 negatively affected host survival against oral infection. This was associated with diminished recruitment of CD4^+^ T cells to the lamina propria (LP), decreased T cell IFN-γ secretion, impaired inflammatory monocyte effector function, and inability to control the parasite in the intestinal mucosa. Reconstitution with CXCR3-competent CD4^+^ T cells restored inflammatory monocyte function, resulting in improved survival against the parasite. Protective effects of adoptively transferred CD4^+^ T lymphocytes depended upon their ability to produce IFN-γ, but occurred independently of CD4 expression of CD40L. Our data show that CXCR3 enables Th1 recruitment to the intestinal LP, where these cells instruct activation of CCR2-dependent inflammatory monocytes, in turn controlling infection. These results establish CXCR3 as a major determinant orchestrating communication between effectors of innate and adaptive immunity, enabling effective host defense against infection.

## Results

### CXCR3 and its ligands CXCL9 and CXCL10 are upregulated during acute toxoplasmosis

**Figure 1:**
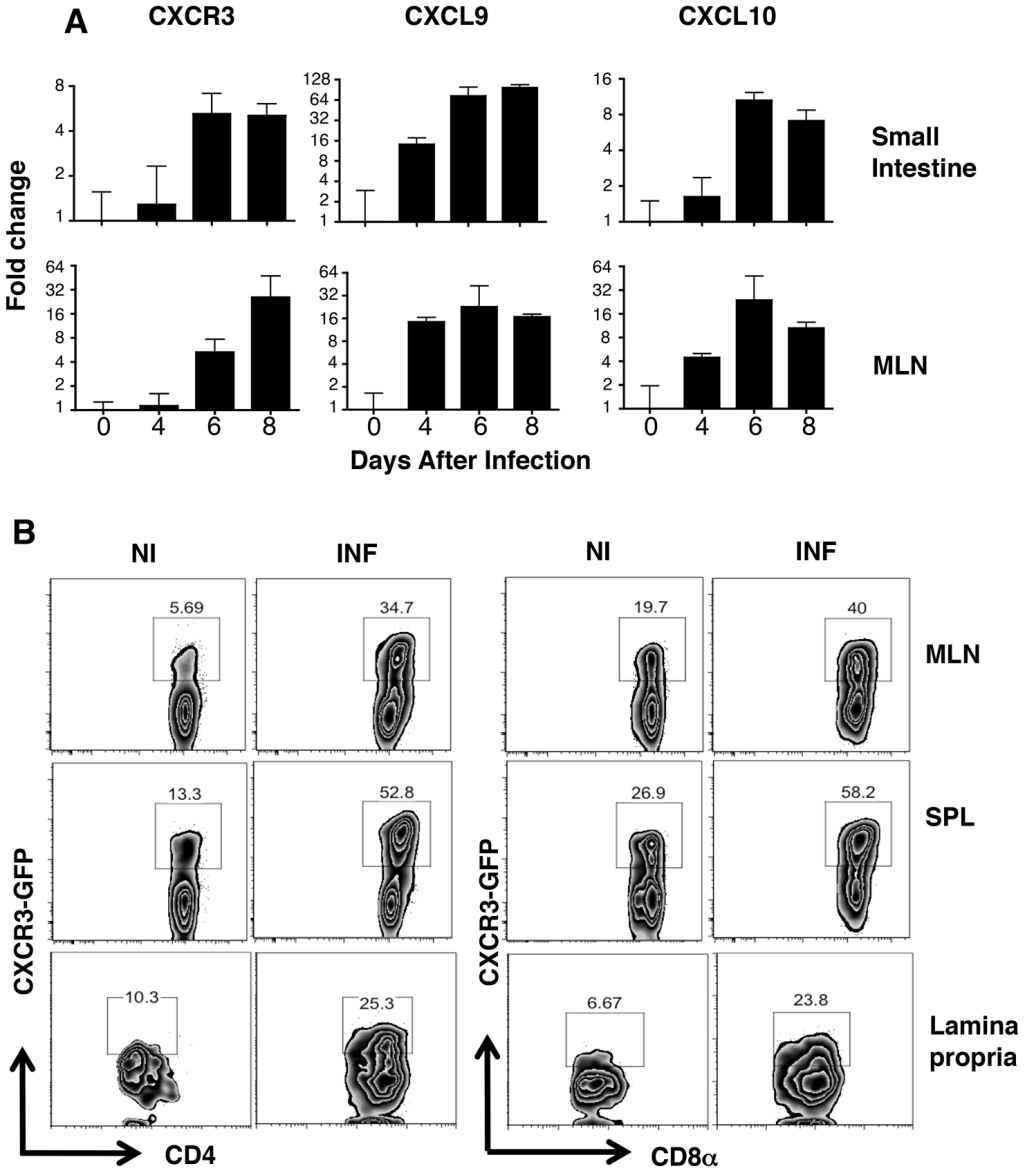
CXCR3 and its ligands are upregulated following *T. gondii* infection. (A) CXCR3, CXCL9, and CXCL10 gene expression was assessed by semi-quantitative real time PCR in mesenteric lymph nodes (MLN) and small intestinal tissue from WT mice during oral *T. gondii* infection. The results are expressed as fold change relative to tissues from noninfected animals (n = 4 mice per time point). (B) CXCR3 protein expression was quantified using flow cytometry by measuring GFP levels before (noninfected, NI) and 11 days after oral infection (INF) of CIBER mice. GFP levels were assessed among CD4^+^ and CD8^+^ T-cell subsets from MLN, spleen (SPL), and small intestinal lamina propria. NI, noninfected; INF, infected.

Because CXCR3 and its chemokine ligands are strongly associated with Th1 responses, we asked whether this proinflammatory axis was induced during *Toxoplasma* infection in the intestinal mucosa. Accordingly, mice were orally inoculated with cysts, and relative levels of CXCR3, CXCL9 and CXCL10 mRNA expression were measured over the course of acute infection. We found strong upregulation of CXCR3 and its specific chemokine ligands as early as Day 4 post-infection in both the ileum and mesenteric lymph nodes (MLN) (Fig. 1A). Overall, peak CXCR3 mRNA levels were attained by Day 6 post-inoculation.

In order to examine CXCR3 expression in more detail, we utilized *Cxcr3* eGFP reporter (CIBER) mice, a bicistronic reporter strain in which cells expressing CXCR3 also express eGFP [21]. We found a large increase in CXCR3 populations of both CD4^+^ and CD8^+^ T lymphocytes in MLN, spleen (SPL) and LP compartments following infection (Fig. 1B). In general, CXCR3 upregulation was most pronounced in the CD4^+^ population. For example, in the MLN there was a 6-fold increase in CD4^+^CXCR3^+^ cells but only a 2-fold increase in CD8^+^CXCR3^+^ lymphocytes. NK cells are also known to express CXCR3 and are an important source of early IFN-γ during *T. gondii* infection [22], [23]. However, while CXCR3-GFP expression was relatively high on naïve NK cells, the GFP expression was in fact reduced during infection, suggesting lack of a role for CXCR3^+^ NK cells during intestinal infection (Fig. S1). We next examined expression of the activation marker CD27 on GFP^+^ and GFP^−^ T lymphocytes in infected mice. CD27 was significantly lower in CXCR3^−^GFP^−^ cells, suggesting an altered maturation state of the CXCR3^−^ T cells (Fig. S2A and B). Likewise, there was a lower percentage of CD27^+^ cells amongst CXCR3-GFP^−^ CD8^+^ T lymphocytes, although the decreases in expression were not as striking as with the CD4^+^ lymphocytes (Fig. S2C and D).

### *Cxcr3^−/−^* mice are increased in susceptibility and are prone to severe intestinal damage following *T. gondii infection*

**Figure 2:**
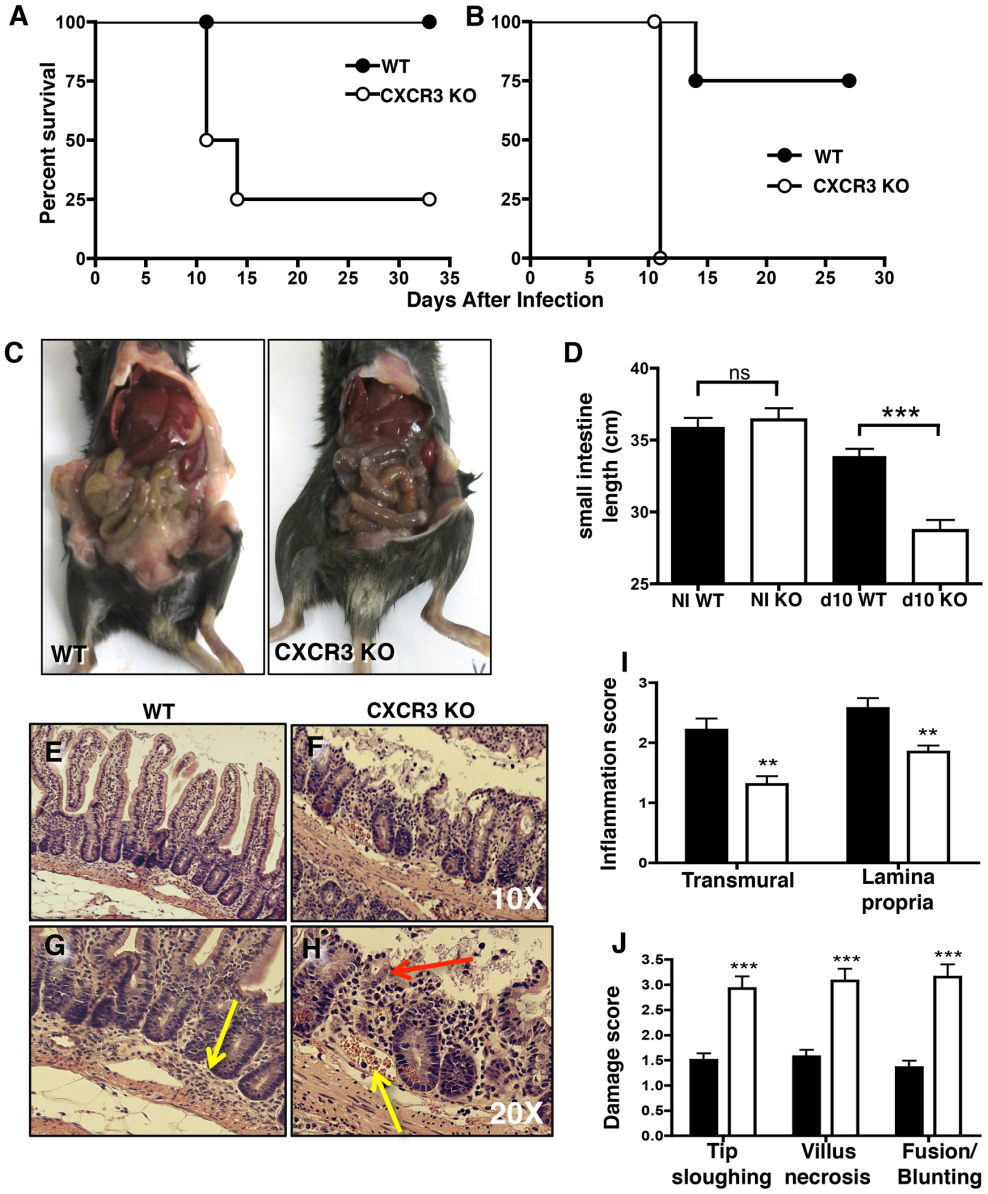
*Cxcr3^−/−^* mice are susceptible to severe intestinal pathology following oral *T. gondii* infection. WT and CXCR3-deficient mice were orally inoculated with 30 ME49 cysts (A) or 50 cysts (B) of *T. gondii* and monitored for survival. In another set of experiments (C–J), mice were orally inoculated with 30 ME49 cysts, and tissues were collected at Day 10 post-infection. (C) Gross intestinal lesions in representative WT and CXCR3 KO mice. (D) Average lengths of noninfected (NI) and infected (INF) WT and *Cxcr3* ^−/−^ small intestines (NI WT, n = 5; NI KO, n = 3; INF WT, n = 8, INF KO: n = 7). E–H, H&E stained sections of small intestines from infected WT (E and G) and KO (F and H) mice. In panel G, the arrow points to an area of inflammatory cell influx. In panel H, the yellow arrow indicates an area of vascular congestion, and the red arrow indicates a necrotic villus. Blind scoring was performed on H&E stained intestine sections for inflammation (I) and damage (J) criteria (WT: n = 14; KO: n = 13; * p<0.05, ** p<0.01, *** p<0.001). Pooled data are represented as mean +/− SEM.

To further examine the role of CXCR3 during *T. gondii* infection, mice deficient in CXCR3 were orally inoculated with low virulence ME49 cysts, and the outcome of infection was monitored. While all wild-type (WT) mice survived acute infection with 30 cysts, *Cxcr3^−/−^* animals displayed increased susceptibility with nearly 75% of mice dying by 2 weeks post-infection (Fig. 2A). When the cyst dose was increased to 50, all CXCR3 knockout (KO) mice rapidly succumbed to infection, but some WT mice also died (Fig. 2B). Interestingly, when WT and KO mice were infected by intraperitoneal injection, lack of CXCR3 did not affect survival, indicating that the effect of CXCR3 is specific to the mucosal response (Fig. S3A). To further examine the overall response in orally infected mice, we examined the gross appearance of the small intestine of WT and *Cxcr3^−/−^* mice after 30-cyst infection. The small intestines of the KO mice were strikingly damaged as demonstrated by massive hemorrhage compared to WT (Fig. 2C). Consistent with intestinal shortening associated with increased damage [24]–[26], the length of the small intestine was reduced in the KO mice during infection (Fig. 2D). Increased damage was further confirmed by H&E staining of small intestinal sections. WT mice displayed minor villus blunting accompanied by moderate to severe inflammatory cell recruitment in the submucosa (Fig. 2E and G). In contrast, *Cxcr3^−/−^* mice displayed severe villus blunting, fusion, epithelial necrosis, sloughing of villus tips, and vascular congestion and hemorrhage (Fig. 2F and H). Blind scoring of H&E sections revealed a significant decrease in inflammation scores in the absence of CXCR3 (Fig. 2I), but when parameters of intestinal damage were quantitated, *Cxcr3^−/−^* mice scored significantly higher than WT counterparts (Fig. 2J). This damage was infection-dependent as intestines from non-infected WT and *Cxcr3^−/−^* mice both had normal architecture with few inflammatory cells (Fig. S3B). Increased epithelial damage in the absence of CXCR3 was further verified by loss of epithelial surface-associated Muc1 compared to infected WT animals, suggesting epithelial cell sloughing (Fig. S3C). Despite the overall decreased inflammatory score, *Cxcr3^−/−^* mice consistently displayed an influx of neutrophils into the LP compartment compared to WT mice, suggesting a role for these cells in causing damage, as argued by others [13], [14], [27] (Fig. S3D and E).

### *Cxcr3^−/−^* mice are unable to control parasite replication in the small intestine

**Figure 3:**
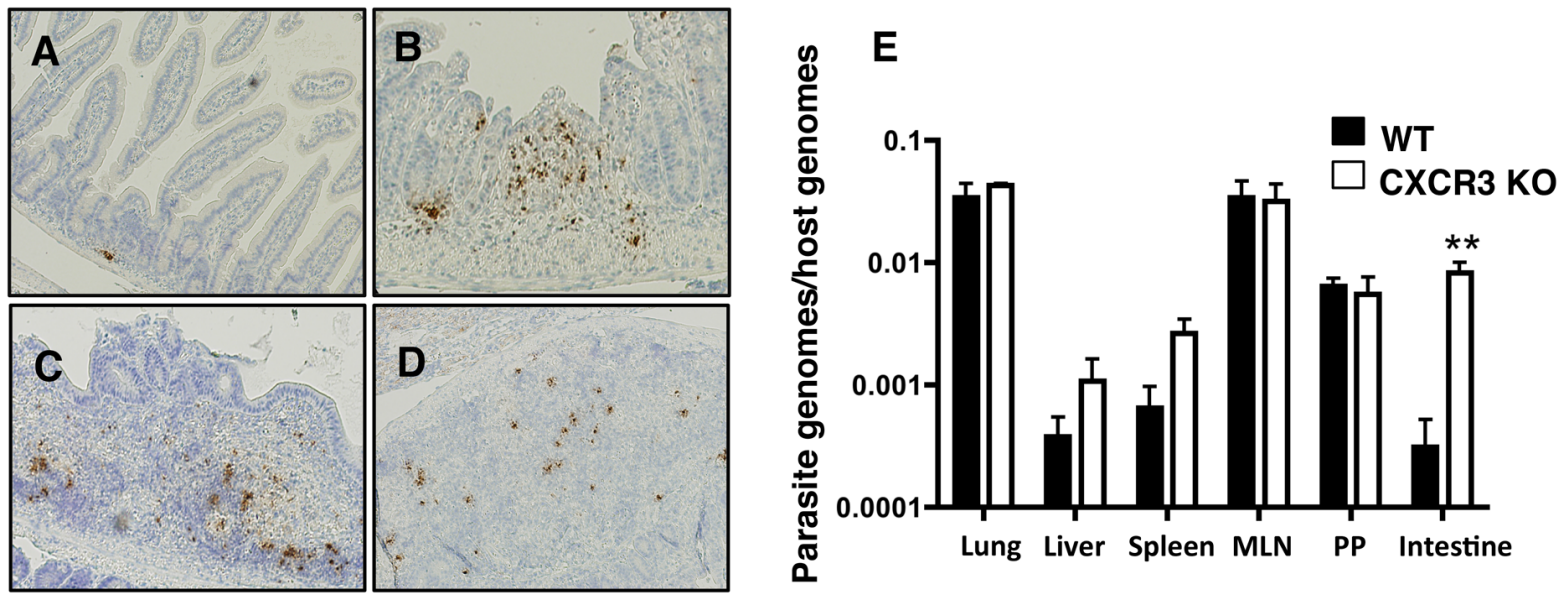
Intestinal parasite burden is elevated in *Cxcr3^−/−^* mice. Paraffin sections of Day 10-infected WT and *Cxcr3^−/−^* small intestines were stained by immunohistochemistry for *T. gondii* antigen. Shown are representative images of WT LP (A), *Cxcr3^−/−^* LP (B), WT Peyer's patch (C), and *Cxcr3^−/−^* Peyer's patch (D) with positive parasite staining in brown. (E) Lung (WT: n = 3; KO: n = 4), liver (WT: n = 5; KO: n = 4), spleen (WT: n = 5; KO: n = 4), MLN (WT: n = 3; KO: n = 5), Peyer's patches (PP, WT: n = 2, KO: n = 2) and small intestines (WT: n = 5; KO: n = 4) were harvested during acute infection, and DNA was isolated from tissues and subjected to quantitative PCR amplification for the parasite B1 gene and the host arginosuccinate lyase gene. Parasite burden was quantitated as parasite to host genome equivalents and was calculated by comparison to a standard curve obtained from known amounts of *Toxoplasma*. Pooled ratios are represented as mean +/− SEM where **p<0.01.

Genetic knockout of cytokines such as IFN-γ results in susceptibility to *T. gondii* through the inability to control parasite replication, whereas the deletion of anti-inflammatory mediators such as IL-10 results in susceptibility due to cytokine pathology [28], [29]. Based on decreased inflammation scores, we hypothesized that the *Cxcr3^−/−^* mice were more likely to be succumbing from uncontrolled parasite replication rather than immune-mediated damage. To examine this, intestinal tissues were stained for parasite antigen by immunohistochemistry. Sections from WT mice displayed minimal parasite infiltration within the LP (Fig. 3A). Conversely, *Cxcr3^−/−^* mice contained numerous large foci of parasite throughout the length of the small intestine that often coincided with areas of severe damage (Fig. 3B). Surprisingly, this difference was restricted to the LP and submucosa of the small intestine because Peyer's patches (PP) in WT and *Cxcr3^−/−^* mice contained similar levels of parasite antigen (Fig. 3C and D). Differences in parasite burden between WT and *Cxcr3^−/−^* mice in the MLN, spleen, and lung were also indiscernible by IHC analysis (data not shown). These results were further confirmed by quantitative PCR. Thus, while lung, liver, spleen, MLN and PP contained similar levels of parasite genomes regardless of CXCR3 expression, there was an approximately 50-fold increase in parasite levels in the absence of CXCR3 in intestinal tissues (Fig. 3E). These data suggest that increased susceptibility to *Toxoplasma* in *Cxcr3^−/−^* mice was due to a localized inability to control parasite replication within the LP of the small intestine.

### CD4^+^ T cells are recruited to the small intestine via CXCR3

**Figure 4:**
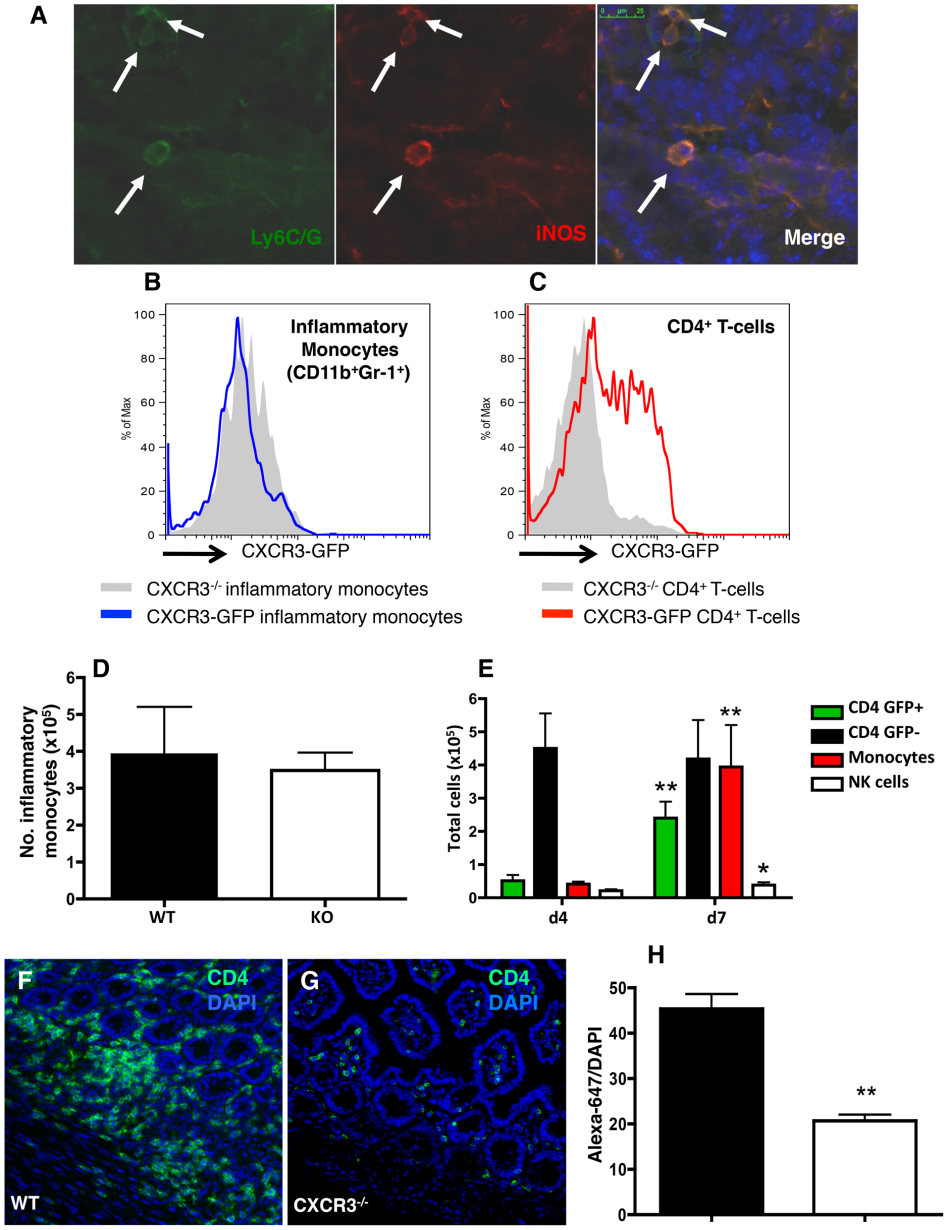
CD4^+^ T-cell recruitment, but not the presence of inflammatory monocytes, is impaired in the small intestine in the absence of CXCR3. (A) Frozen sections of intestines from infected WT mice were co-stained for Ly6C/G (Gr-1) (green) and iNOS (red) to confirm the presence of inflammatory monocytes in the mucosa of Day 6-infected animals. (B and C) Small intestinal LP cells were isolated from CXCR3-GFP reporters and *Cxcr3^−/−^* mice 6 days following oral infection. In the CXCR3 reporter mice, inflammatory monocytes (B, blue line) and CD4^+^ T cells (C, red line) were assessed for GFP expression by flow cytometry as compared to *Cxcr3^−/−^* cells (gray shaded in both histograms). (D) Total numbers of lamina propria inflammatory monocytes 6 days after infection. Neutrophils were excluded by gating on Ly6G-negative cells. (E) Total numbers of CD4^+^CXCR3-GFP^+^ T cells, CD4^+^CXCR3-GFP^−^ T cells, inflammatory monocytes, and NK cells in the lamina propria of WT and *Cxcr3^−/−^* mice 4 and 7 days post-infection. Statistical comparisons were made between time points of respective cell types, where * p<0.05 and ** p<0.01. In panels F–G, WT (F) and *Cxcr3^−/−^* (G) intestinal frozen sections were stained with anti-CD4 antibody followed by anti-rat Alexa-647. Sections were visualized by immunofluorescence microscopy. (H) To quantify CD4^+^ T-cell infiltration, the ratio of Alexa-647 over DAPI fluorescence was calculated (WT: n = 3; KO: n = 3; 6–12 fields/mouse; p<0.01). Pooled ratios are represented as mean +/− SEM.

The dominant effector cells required for elimination of *T. gondii* following oral infection are inflammatory monocytes. These cells express Ly6C/G (Gr-1), produce TNF-α, IL-12, and are likely to kill parasites via activation of IFN-γ-inducible p47 GTPases that assemble at the parasitophorous vacuole membrane and mediate its destruction [8], [11]. Consistent with others [11], we observed these cells in the LP of infected mice (Fig. 4A). Inflammatory monocytes are dependent upon CCR2 for exit from the bone marrow, but we wondered whether CXCR3 might be involved in recruiting these cells to the LP in response to *T. gondii*. Therefore, we examined CXCR3-GFP expression by intestinal inflammatory monocytes during infection. Inflammatory monocytes in the small intestinal LP of infected reporter mice did not express any GFP as compared to inflammatory monocytes isolated from infected *Cxcr3^−/−^* mice (Fig. 4B). In stark contrast, approximately 50% of LP CD4^+^ T cells expressed high levels of GFP (Fig. 4C). Furthermore, *Cxcr3^−/−^* mice displayed unaltered total numbers of LP inflammatory monocytes compared to wild-type controls (defined as CD11b^+^Ly6C^+^Ly6G^−^) (Fig. 4D). We next assessed the kinetics by which CD4^+^ T cells and inflammatory monocytes were recruited to the lamina propria during infection. Between days 4 and 7 of infection, there was a significant increase in the total numbers of CD4^+^CXCR3^−^GFP^+^ T cells and inflammatory monocytes (Fig. 4E). However, the total number of CD4^+^CXCR3^−^GFP^−^ cells remained unchanged, further indicating that infection promotes the recruitment of CD4^+^CXCR3^+^ T cells (Fig. 4E). Few NK cells were observed in the lamina propria, but there was a small increase in their number during infection. This was attributable to an increase in CXCR3^−^ NK cells (data not shown). Consistent with these results, there was an influx of CD4^+^ T cells in WT small intestines that was diminished in *Cxcr3^−/−^* mice (Fig. 4F–H). These findings demonstrate that CD4^+^ T cells fail to effectively traffic to the intestinal compartment in the absence of CXCR3, but the presence of LP inflammatory monocytes does not require this chemokine receptor.

### Lamina propria CD4^+^ T cells display impaired IFN-γ production

**Figure 5:**
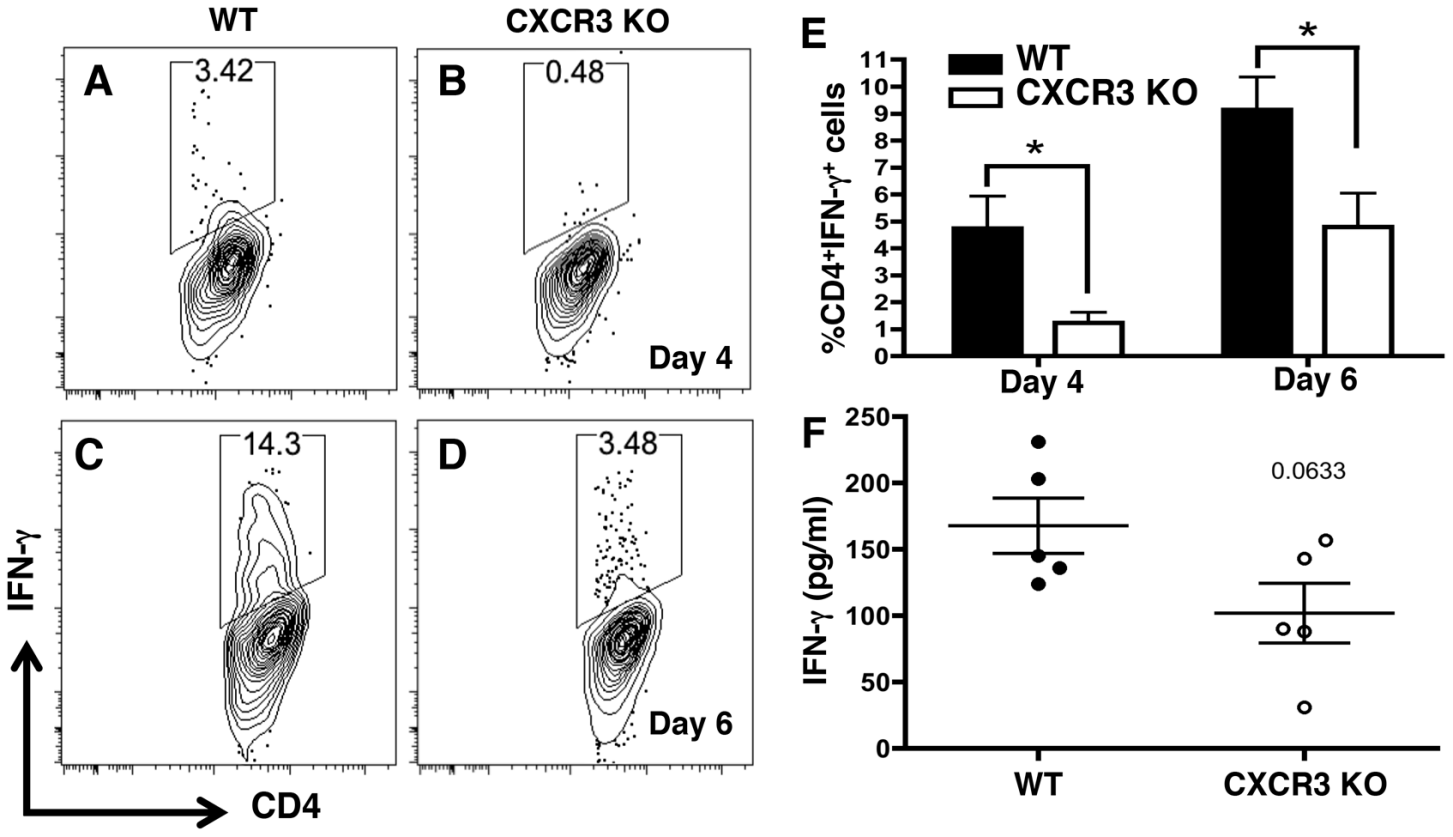
Lamina propria CD4^+^ T cells display impaired IFN-γ production. Lamina propria leukocytes were harvested from WT and *Cxcr3^−/−^* mice at Day 4 (A–B) and Day 6 (C–D) post-infection, cultured in the presence of PMA, ionomycin, and Brefeldin-A for 6 hrs, and assessed for IFN-γ production by flow cytometry. The means and standard errors of individual mice are shown in E (Day 4, n = 10 per strain; Day 6 n = 6 per strain). Intestinal biopsy cultures were performed at Day-6 post-infection, and IFN-γ was measured in the supernatants after 24 hr of culture (F). Each dot represents an individual mouse, and *p<0.05.

We next asked if expression of CXCR3 affected the ability of T cells to secrete the Th1 cytokine IFN-γ. Initial experiments on bulk populations of splenocytes and mesenteric lymph node (MLN) cells from Day-11 infected WT and KO revealed no differences in the amount of IFN-γ, TNF-α or IL-10 produced during *in vitro* culture (Fig. S4). To specifically examine functional outcomes in intestinal cells, WT and *Cxcr3^−/−^* lamina propria leukocytes were harvested 4 and 6 days post-oral infection, stimulated *ex vivo*, and IFN-γ production was examined by flow cytometry. CD4^+^ T cells from both WT and *Cxcr3^−/−^* displayed enhanced IFN-γ production over time. However, in the absence of CXCR3, CD4^+^ T cells produced significantly less IFN-γ compared to WT cells at both examined time points (Fig. 5A–E). This was confirmed by measuring IFN-γ from the supernatants of Day-6 WT and *Cxcr3^−/−^* intestinal biopsy cultures, where IFN-γ was lower in the absence of CXCR3 (Fig. 5F). This effect was specific to the CD4^+^ T cell subset, as IFN-γ production by lamina propria CD8^+^ T cells and NK cells was unchanged between WT and knockout animals (Fig. S5A–F). Further confirming that this loss of IFN-γ production was specific to CD4^+^ T lymphocytes in the small intestine, and consistent with the bulk splenocyte culture experiments, splenocytes isolated from infected WT and *Cxcr3^−/−^* mice secreted equivalent levels of IFN-γ upon *ex vivo* stimulation with PMA and ionomycin (Fig. S5G–I). Together, these results indicate an intestine-specific defect in presence of CD4^+^ Th1 cells in the absence of CXCR3 as measured by the capacity to produce IFN-γ.

### Inflammatory monocyte responses in the intestinal mucosa are defective in the absence of CXCR3

**Figure 6:**
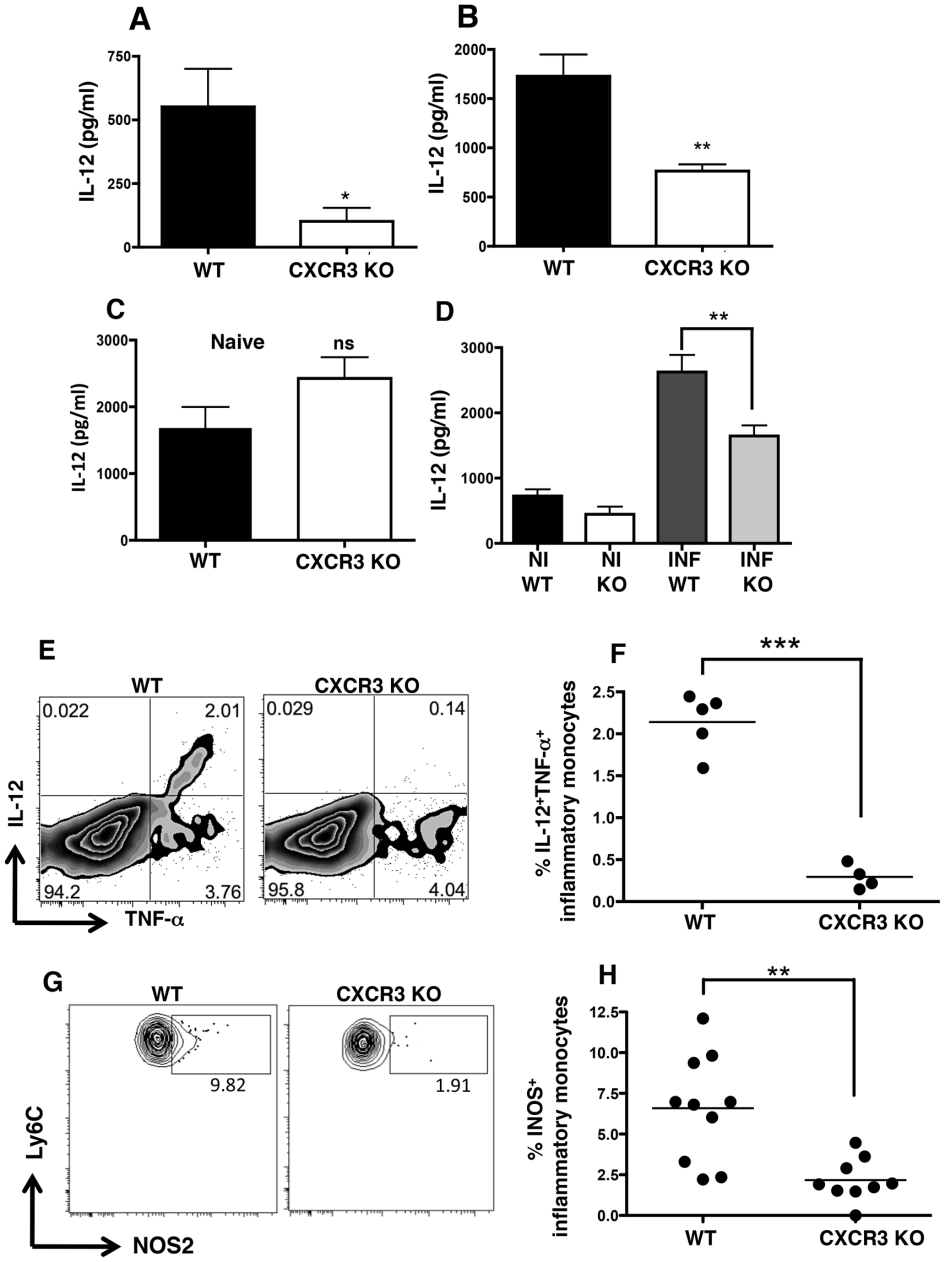
Inflammatory monocyte function is impaired in the absence of CXCR3. MLN cells (A) and splenocytes (B) from Day 11-infected WT and *Cxcr3^−/−^* mice were cultured for 72 hours in complete DMEM in the presence of soluble tachyzoite antigen (STAg) (n = 5 mice per strain). Supernatants were then collected and assayed for IL-12p40 by ELISA. (C) Naïve splenocytes were cultured with soluble tachyzoite lysate for 48 h, and culture supernatants were assayed for IL-12p40 secretion. (D) Intestinal biopsy samples from noninfected (NI) and Day 9-infected (INF) WT and *Cxcr3^−/−^* mice were cultured overnight, then supernatants were collected and assayed for IL-12p40. (WT: n = 18; KO: n = 16). Data are represented as mean +/− SEM. (E) Lamina propria leukocytes from Day 9-infected WT and *Cxcr3^−/−^* mice were cultured in the presence of Brefeldin-A for 6 hr. Cells were surface stained for CD11b, Ly6G (1A8), and Ly6C/G (Gr-1) then intracellularly stained for IL-12 and TNF-α. The cell populations shown are gated on CD11b^+^Gr-1^+^1A8^−^ cells, and the quadrants represent proportions of cells positive for each cytokine. (F) IL-12^+^TNF-α^+^ inflammatory monocyte levels in the lamina propria of individual mice. Each dot represents results from a single mouse. (G) Leukocytes were isolated from the MLN of Day 4-infected WT and *Cxcr3^−/−^* animals and stained for iNOS by flow cytometry. (H) The means of multiple mice are plotted (WT: n = 10; KO: n = 9). In this figure, * p<0.05, ** p<0.01, *** p<0.001.

Although IFN-γ, IL-10, and TNF-α responses remained intact in the MLN and spleen late during infection of CXCR3-deficient mice, a significant decrease in IL-12 production was observed in the MLN (Fig. 6A) and spleen (Fig. 6B) of *Cxcr3^−/−^* mice. Defective IL-12 responses in the CXCR3 KO strain were infection dependent, because parasite antigen stimulated equivalent amounts of IL-12 in noninfected WT and KO splenocytes (Fig. 6C). This response, known to derive from resident splenic CD8α^+^ DC [30], may account for equivalent Th1 priming in secondary lymphoid organs, despite lower IL-12 levels during late acute infection.

Since IL-12 is also a characteristic cytokine of inflammatory monocytes, we investigated the impact of CXCR3 deletion on intestinal inflammatory monocyte function. Indeed, *in vitro* culture of intestinal biopsy samples revealed decreased production of IL-12 (Fig. 6D). To further identify the source of the defective IL-12, we examined the production of IL-12 from inflammatory monocytes. While the total numbers of LP inflammatory monocytes were equivalent in WT and CXCR3 KO mice (Fig. 4B), the population of CD11b^+^Gr-1^+^ cells co-expressing IL-12 and TNF-α was dependent upon CXCR3 (Fig. 6E and F). Further confirming impaired inflammatory monocyte function, iNOS expression was significantly decreased in inflammatory monocytes (Fig. 6G and H). These findings strongly suggest that inflammatory monocytes are functionally impaired in the absence of CXCR3. Interestingly, neutrophils in the LP of KO mice produced significantly higher levels of TNF-α compared to WT neutrophils (Figure S6A and B).

### Adoptive transfer of WT CD4^+^ T lymphocytes rescues inflammatory monocyte function and restores resistance in *Cxcr3^−/−^* mice

**Figure 7:**
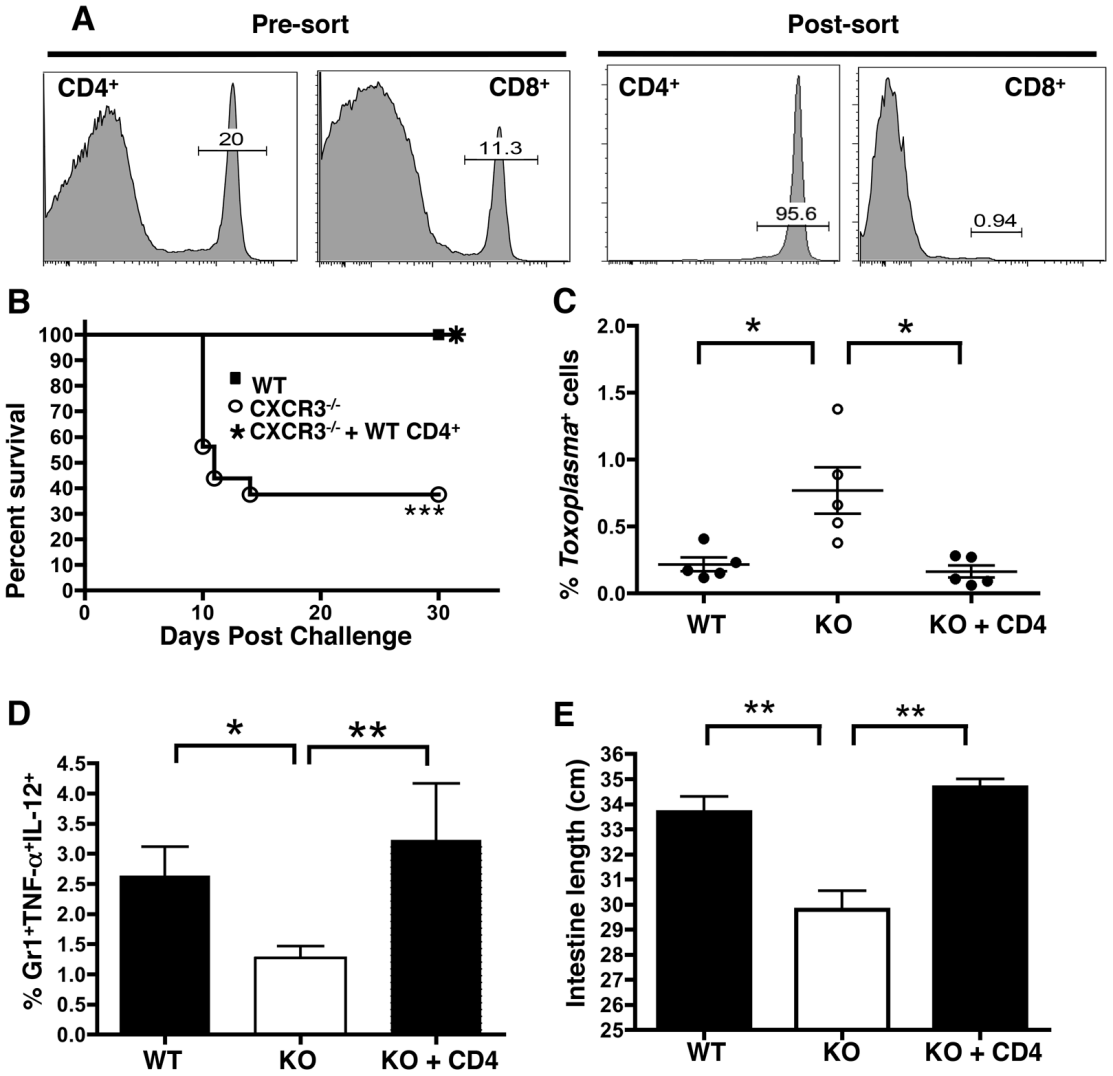
Adoptive transfer of WT CD4^+^ T cells into *Cxcr3^−/−^* mice confers resistance to infection. (A) Splenocytes from naïve WT mice were sorted for CD4^+^ T cells by magnetic bead separation. Pre-sort and post-sort fractions were stained for CD4 and CD8 to confirm efficacy of the sort. (B) Purified CD4^+^ T cells (5×10^6^) from noninfected mice were adoptively transferred by intravenous injection into *Cxcr3^−/−^* recipients (n = 5 mice per group). Mice were orally challenged 24 hr post-transfer with *T. gondii* (30 cysts), and survival was monitored. ***, p<0.001 comparing *Cxcr3^−/−^* with *Cxcr3^−/−^*+CD4^+^ T cells. (C–E) Lamina propria leukocytes were isolated from WT, *Cxcr3^−/−^* controls, and *Cxcr3^−/−^* CD4^+^ T-cell recipients at Day 9 post-infection (n = 5 mice per group). (C) Total cells were stained for *Toxoplasma* surface antigen (SAG)-1 to determine parasite infection. (D) Cells were also cultured in the presence of Brefeldin-A for 6 hours, after which they were surface stained for CD11b, Gr-1, and Ly6G and stained intracellularly forIL-12 and TNF-α. (E) Small intestinal length was compared between WT, *Cxcr3^−/−^*, and *Cxcr3^−/−^* adoptive transfer recipient mice (WT: n = 11; KO: n = 10; Transfer: n = 3). Data are represented as mean +/− SEM, where * p<0.05 and ** p<0.01.

Given the data so far, we hypothesized that CD4^+^ T cells were unable to effectively home to the small intestine and prime inflammatory monocyte function in the absence of CXCR3, resulting in susceptibility to *Toxoplasma*. We therefore tested whether reconstitution with CXCR3-competent CD4^+^ T cells would allow *Cxcr3^−/−^* mice to overcome susceptibility and restore inflammatory monocyte function. Accordingly, CD4^+^ T cells from naïve WT spleens were enriched to 90–95% purity by magnetic bead separation (Fig. 7A) and injected i.v. into *Cxcr3^−/−^* recipients. Mice were orally challenged with *T. gondii* 24 hours post-adoptive transfer, and survival was monitored. Knockout mice that did not receive WT cells began to die 10 days post-challenge, while all KO mice that received CD4^+^ T cells and all WT controls survived the acute phase of infection (Fig. 7B). To confirm that the CD4-dependent survival was not an artifact of the transfer, *Cxcr3^−/−^* CD4^+^ T cells were adoptively transferred into KO recipients. The knockout cells were unable to protect against susceptibility, demonstrating that protection is dependent on CXCR3 expression by CD4^+^ T cells (Fig. S7A).

To assess the functional impact of WT CD4 adoptive transfer, parasite burden and cytokine production in the LP were assessed by flow cytometry in WT, *Cxcr3^−/−^*, and *Cxcr3^−/−^* +CD4 mice. While *Cxcr3^−/−^* mice had a clear increase in *Toxoplasma* infected cells relative to WT, upon adoptive transfer of WT CD4^+^ T cells, parasite levels were reduced to WT (Fig. 7C). Likewise, expression of IL-12/TNF-α by inflammatory monocytes was significantly reduced in KO mice, but these cytokines returned to WT levels upon transfer of WT CD4^+^ T cells (Fig. 7D and Fig. S7B). In addition to cytokine responses, intestinal damage was also alleviated by adoptive transfer of WT CD4^+^ T cells, as the intestinal lengths of the transferred mice were restored to WT (Fig. 7E). Possibly as a result of improved monocyte function and parasite clearance, neutrophil levels and neutrophil TNF-α secretion were also restored to WT levels following the transfer of WT CD4^+^ T cells (Fig. S7C and D).

### CD4+ T-cell rescue is dependent on IFN-γ but independent of CD40L

**Figure 8:**
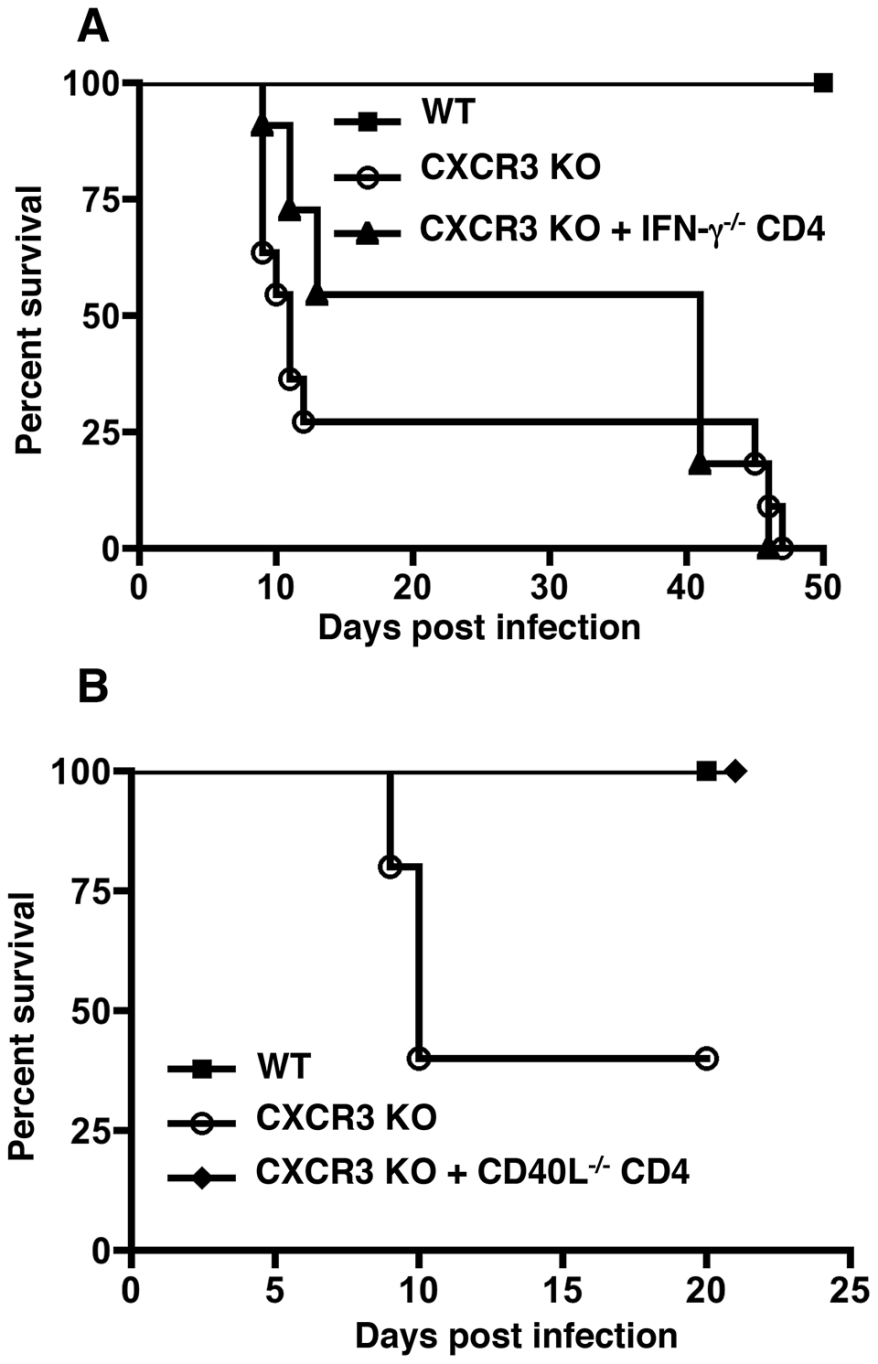
CD4-mediated rescue is dependent on IFN-γ but independent of CD40L. CD4+ T cells were isolated from *Ifn-γ^−/−^* (A) and *Cd40l^−/−^* (B) mice, adoptively transferred into *Cxcr3^−/−^* mice that were subsequently challenged with *Toxoplasma* as described in the Figure 6 legend (WT: n = 10; KO: n = 11; *Ifn*-γ*^−/−^*: n = 11; *CD40L^−/−^*: n = 5).

To identify the mechanism behind the rescue of *Cxcr3^−/−^* susceptibility by WT CD4^+^ T cells, we performed adoptive transfer experiments utilizing T cells derived from knockout animals. Inasmuch as CXCR3 is a Th1 chemokine receptor, we began by asking whether reversal of susceptibility was dependent on CD4-derived IFN-γ Therefore, CD4^+^ T cells were isolated from *Ifnγ* mice and adoptively transferred into CXCR3-deficient recipients. Unlike IFN-γ-competent CD4^+^ T cells (Fig. 7B and Fig. 8B), transfer of IFN-γ KO CD4^+^ T lymphocytes failed to provide significant protection (Fig. 8A). It has been shown that CD40L contributes to inflammatory responses in the intestinal mucosa during oral *Toxoplasma* infection [31]. Therefore, we performed the adoptive transfer using *Cd40l^−/−^* CD4^+^ T cells and assessed survival. As expected, CXCR3-deficient animals were highly susceptible to infection. However, *Cxcr3^−/−^* mice receiving *Cd40l^−/−^* CD4^+^ T cells survived the infection, indicating that CD40L does not mediate CXCR3^+^CD4^+^-dependent protection (Fig. 8B).

## Discussion

Effective control of pathogens such as *T. gondii* requires the coordinated action of cells of innate and adaptive immunity. Orchestration of the response is governed by an underlying network of chemokines and chemokine receptor-expressing cells in both the hematopoietic and non-hematopoietic compartments. In this study, we demonstrate a central role for chemokine receptor CXCR3 in empowering Th1 trafficking to the small intestine, in turn enabling inflammatory monocyte activation and concomitant control of infection. While the importance of IFN-γ-secreting CD4^+^ T cells in resistance to *Toxoplasma* is well known to researchers in the field [28], [32], [33], and while the importance of anti-microbial inflammatory monocytes has recently become clear in the context of *Toxoplasma* and other infections [11], the present study is the first to reveal the functional link between CXCR3^+^ T-cell effectors, IFN-γ and inflammatory monocyte activation in tissues of the intestinal mucosa.

Although Th1 effectors depend upon CXCR3 to reach the site of infection, inflammatory monocytes require chemokine receptor CCR2 for optimal trafficking. In the latter case, inflammatory monocytes fail to exit the bone marrow in *Ccr2^−/−^* mice, resulting in a decreased level of this population in the periphery, in turn resulting in inability to control *T. gondii* infection [11]. Monocytes have also been suggested to promote the systemic dissemination of *T. gondii* to the brain [34]. While brain parasite loads were not examined in this study, it is unlikely that altered parasite shuttling is a mechanism by which the knockout animals are succumbing to *Toxoplasma* because peripheral parasite loads were not affected by absence of CXCR3 (Fig. 3B). Our data argue that the basis for increased parasites specifically in the intestine is the result of defective regional control by inflammatory monocytes that lack CXCR3-dependent activation signals. Recent data indicate that inflammatory monocyte expression of CCR1 enables a response to IL-15-dependent CCL3-secreting innate lymphoid cells, resulting in CCR1-dependent recruitment to the intestinal mucosa of *Toxoplasma* infected mice [35]. Taking these data and ours collectively, we propose that CXCR3, CCR2 and CCR1 act together as a control axis of innate and acquired immunity in intestinal immunity, ensuring coordinated recruitment of inflammatory monocytes and Th1 effectors to inflamed tissues.

It was recently demonstrated that NK cell-derived IFN-γ controls the differentiation of circulating monocytes into inflammatory dendritic cells during i. p. *T. gondii* infection, and is thus required for an optimal IL-12 response [36]. In our model we did not see a dependence on CXCR3 for NK cell recruitment, as NK cells appeared to lose CXCR3-GFP expression over the course of infection (Fig. S1), and production of NK cell IFN-γ was equivalent in WT and KO mice (Fig. S5D–F). This difference may be attributable to the alternative routes of infection used, as our model incorporates the intestinal response, while the i.p. route bypasses the intestinal mucosa. Consistent with this idea, absence of CXCR3 did not affect the ability of mice to survive i. p. infection (Fig. S3A).

While our study focuses on the early response to *Toxoplasma* infection in the intestinal mucosa, others have examined the role of CXCR3 and its ligands in additional tissues and at different stages of infection. Antibody-mediated depletion of CXCL10, a major CXCR3 ligand, increases susceptibility and blocks influx and expansion of T cells in the liver and spleen that accompanies *T. gondii* infection [37]. Additionally, a study of ocular toxoplasmosis revealed that T cells infiltrating the eye during infection express CXCR3 and produce IFN-γ. Depletion of CXCL10 in this model reduced the number of infiltrating T lymphocytes during chronic infection, resulting in increased parasite replication and ocular damage [38]. Recently, CXCL10 was shown to impact CD8^+^ T-cell mobility in the brain of chronically infected mice, enhancing their ability to control the parasite by increasing contact with infected cells [39]. Our results for the first time highlight the importance of CXCR3 and its impact on CCR2-dependent monocytes in the initial protective response to the parasite in the intestine.

It has been shown that TGF-β production by intestinal IEL protects against *T. gondii*-induced damage by down-modulating inflammation [40]. While we did not examine intraepithelial lymphocytes (IEL) in this study, it is possible that CXCR3 expression could also affect trafficking and function of this cell type, thereby contributing to resistance in this model. Future studies will allow us to determine whether CXCR3-expressing IEL play a role in immunity during intestinal *T. gondii* infection.

CXCR3 has also been assessed for its role in immunity to other protozoan pathogens, including *Leishmania* and *Plasmodium*. Interestingly, the function of CXCR3 differs depending upon the parasite, the route of infection and the site examined. For example, *Cxcr3^−/−^* mice exhibit impaired IFN-γ production and increased lesion development during cutaneous *L. major* infection, but the knockout mice are not more susceptible to hepatic *L. donovani* infection [19], [41]. Furthermore, CXCR3 and its chemokines promote cerebral inflammation and mortality during experimental malaria infection [20], [42]. Overall, CXCR3 and its chemokine ligands function as double-edged swords, inasmuch as they make an important contribution to protective immunity, but when dysregulated they are the cause of deleterious immunopathology.

In addition to its role in cell recruitment, CXCR3 has recently been suggested to be important for priming CD4^+^ T cells in the lymph node to become Th1 cells by promoting long-lasting interactions between T cells and CXCL10-expressing dendritic cells. In the absence of CXCR3, T cells fail to fully differentiate into IFN-γ-producing cells and are defective during subsequent lymphocytic choriomeningitis virus (LCMV) infection [43]. We found no evidence for defective Th1 responses in secondary lymphoid organs during *Toxoplasma* infection of *Cxcr3^−/−^* mice, a result that is supported by similar findings during *L. major* infection [19]. However, our results are consistent with impaired recruitment of Th1 cells in the absence of CXCR3, as lamina propria CD4^+^ T cells from *Cxcr3^−/−^* mice exhibited defective IFN-γ production. We conclude that the function of CXCR3 in promoting Th1 differentiation versus, or in addition to, homing to inflammatory sites is likely to be a context-dependent phenomenon.

Although we found no differences in T-cell activation in CXCR3 negative T cells of reporter mice or in CXCR3 KO animals, as measured by expression of CD25, CD69 and CD44 (data not shown), we observed a consistent decrease in CD27 expression amongst CXCR3-negative T cells of reporter mice. Overall levels of CD27 expression were also lower in T cells from *Cxcr3^−/−^* mice (data not shown). CD27 is a member of the TNF receptor super family that has been implicated as a T-cell costimulatory molecule [44]. CD27 expression is thought to characterize naïve or memory T cells, whereas loss of CD27 represents terminal differentiation [45]. Accordingly, it is possible that in addition to controlling T-cell recruitment to sites of infection, *Cxcr3^−/−^* T cells may undergo terminal differentiation and, possibly, premature Th1 effector death in tissues prior to mediating IFN-γ dependent inflammatory monocyte activation. This is consistent with studies showing that CD27^low^ cells are more susceptible to apoptosis and that accumulation of influenza-specific T lymphocytes was impaired in the lungs of *Cd27^−/−^* mice during infection [44], [46]. In our study, CD27 expression was not rescued by adoptive transfer of WT CD4^+^ T cells (data not shown), which may suggest that increasing the T-cell pool allows the system to cross a certain threshold of T-cell levels in order to activate inflammatory monocytes without reversing CD27 expression.

In the absence of CXCR3, we found that lamina propria neutrophil levels were increased during infection, as was their activation status as measured by TNF-α expression. Furthermore, adoptive transfer of WT CD4^+^ T lymphocytes into the KO strain reversed increased levels of PMN as well as their production of TNF-α. Because this inflammatory cytokine has been linked to intestinal damage during *Toxoplasma* infection [47], it seems likely that CXCR3-dependent effects on neutrophils are likely to be secondary to loss of inflammatory monocyte function. In this scenario, defects in monocyte-mediated parasite killing would result in damage to the intestinal mucosa. Translocation of luminal gut flora, known to contribute to emergence of parasite-induced intestinal lesions [48]–[50], would in turn be expected to result in local neutrophil recruitment. Indeed, based on neutrophil depletion studies it has been suggested that these cells mediate damage to the intestinal mucosa during *T. gondii* infection [27]. The cellular and molecular basis for this effect is not at present known, but both the IL-17/IL-23 axis and CXCL8 have been shown to promote neutrophil accumulation in infected tissues suggesting involvement of one or both of these mediators [51]–[53].

The results of this study extend our understanding of immunity in the intestinal mucosa, which has become increasingly important as inflammatory bowel diseases (IBD), such as ulcerative colitis and Crohn's disease, become more common in developed regions of the world. In this regard, abnormally high levels of CXCR3 are associated with dysregulated intestinal responses in human IBD patients, underscoring the potential hazards of unbalanced inflammatory responses [54]–[56]. While CXCR3 can be pathogenic by recruiting effector cells to otherwise healthy tissue, as in IBD or cerebral malaria, we show here that CXCR3-expressing T cells play an essential protective role in host defense by enabling defense against pathogenic organisms. Antibodies against CXCL10 have been suggested as potential therapeutic agents against IBD [57], [58]. The results from this study, however, highlight the possible harm of inhibiting CXCR3-expressing cells into sites of inflammation during infection with an enteric pathogen.

As the first study to demonstrate a protective role for CXCR3^+^CD4^+^ T cells in the intestinal immune response, we have shown here that failure to appropriately recruit these T cells results in impaired inflammatory monocyte activation, accumulation of intestinal parasites, and subsequent recruitment of potentially pathogenic TNF-α-secreting neutrophils. Our results reveal CXCR3 as a critical chemokine receptor of the adaptive immune system that ensures appropriate placement of T cells in inflamed tissue, enabling inflammatory monocytes of innate immunity to acquire effector functions and mediate effective host defense.

## Materials and Methods

### Ethics statement

All experiments in this study were performed strictly according to the recommendations of the Guide for the Care and Use of Laboratory Animals of the National Institutes of Health. The protocols were approved by the Institutional Animal Care and Use Committee at Cornell University (permit number 1995–0057). All efforts were made to minimize animal suffering during the course of these studies.

### Mice and infections

Female Swiss Webster mice (6–8 weeks of age) were purchased from the Jackson Laboratory (Bar Harbor, ME). *Cxcr3^−/−^* and *Cxcr3* eGFP knock-in reporter mice [21] were established as breeding colonies in the Transgenic Mouse Facility at the Cornell University College of Veterinary Medicine. The CXCR3 internal ribosomal entry site bicistronic eGFP reporter (CIBER) mice were generated as described [21]. This strain possesses a functional CXCR3 receptor, and all CXCR3 positive cells also express intracellular eGFP. Mouse infections were initiated by oral inoculation of cysts of the type II *T. gondii* ME49 strain. Cysts were isolated from chronically infected Swiss Webster mice by homogenization of whole brain in sterile PBS. Unless stated otherwise, mice were infected at 8–12 weeks of age with 30 cysts.

### Tissue staining and immunofluorescence microscopy

Intestines were excised, flushed with 10% neutral-buffered formaldehyde, and embedded in paraffin for sectioning. Sections were stained for hematoxylin and eosin for assessment of pathological changes. Sections were also stained for parasite antigen by immunohistochemistry at the Cornell Animal Health Diagnostic Center. Frozen sections were obtained by embedding 1 cm lengths of intestine in OCT. Sections of 6–8 µm were cut on a cryostat, fixed in ice-cold acetone, and blocked with PBS containing 2× casein and goat serum. To examine T-cell infiltration, sections were incubated with rat anti-CD4 antibody (GK1.5) (ATCC, Manassas, VA) or rat IgG at 4°C overnight followed by goat anti-rat Alexa-647 secondary antibody (Life Technologies, Grand Island, NY). Sections were then mounted in DAPI-Prolong antifade (Life Technologies) and imaged by confocal microscopy. ImageJ software was used to analyze fluorescence of independent channels.

### Cell isolation

Spleens and mesenteric lymph nodes were excised, crushed between sterile slides, and passed through a 70-µm filter (BD, Franklin Lakes, NJ). Red blood cells from splenocyte suspensions were lysed with ACK lysis buffer (Life Technologies). For LP leukocyte isolation, the small intestine was removed, cleaned of mesentery, flushed with sterile PBS, and cleared of Peyer's patches. The intestine was opened longitudinally and the mucosal layer was scraped with a blunt scalpel to remove epithelial cells. The tissue was cut into 5 mm sections and vigorously washed with Dulbecco's modified Eagle's media (Cellgro, Manassas, VA) and 5 mM EDTA (Life Technologies). Cells were liberated from the intestinal tissue by digestion with 10 mg/ml collagenase (Sigma, St. Louis, MO) at 37°C and subsequently passed through a 70-µm filter.

### Cytokine measurement

Secretion of IFN-γα, TNF-α, and IL-10 was assayed by ELISA (eBioscience, San Diego, CA) following manufacturer's instructions in the presence of soluble tachyzoite antigen (STAg) prepared as previously described [59]. IL-12p40 was quantitated using an in-house ELISA [60]. For ileum biopsy cultures, 1 cm intestinal sections were flushed with PBS, opened longitudinally, and cultured overnight in complete Dulbecco's modified Eagle's media supplemented with 10% bovine growth serum (Hyclone), 0.05 mM β-mercaptoethanol (Sigma), 1 mM sodium pyruvate, 0.1 mM nonessential amino acids, 10,000 U/ml penicillin, 10,000 µg/ml streptomycin, and 30 mM HEPES (reagents from Life Technologies). Supernatants were collected and assayed for cytokine by ELISA.

### Measurement of mRNA by quantitative PCR

RNA was isolated from MLN and intestinal tissue from mice over a time course of infection. Tissue was initially disrupted with a tissue homogenizer and subjected to RNA isolation following manufacturer's instructions (Tissue RNA Kit, Omega Biotek, Norcross, GA). RNA was converted to cDNA (Quantas Biosciences, Gaithersburg, MD) and assayed for gene expression by SYBR green technology (Quanta Biosciences). Primers were designed to span exons by Integrated DNA Technologies. GAPDH was used as a housekeeping gene. Gene expression from each timepoint was normalized to uninfected control samples.

### Flow cytometry

Single cell suspensions were pelleted and resuspended with primary antibodies (BioLegend, San Diego, CA: anti-CD4 PerCP, anti-CD8α APC-Cy7, anti-CD45 Alexa-488, anti-CD11b APC-Cy7, anti-CD11b APC, anti-Ly6G FITC; eBioscience, San Diego, CA: anti-CD25 PE, anti-CD45 APC or FITC, anti-CD69 PE, anti-Ly6C/G APC; BD Biosciences, San Jose, CA: anti-Ly6G PE-Cy7, anti-Ly6C V420, anti-CD45 PE, anti-Ly6C/G PerCP, anti-CD44 APC) in ice-cold FACS buffer (1% bovine serum albumin/0.01% NaN_3_ in PBS) for 30 min. For IFN-γ staining, cells were incubated for 6 hrs with Brefeldin-A (eBioscience; 10 ug/ml), PMA (Sigma; 10 ng/ml), and ionomycin (Sigma; 1 ug/ml), then fixed with 4**%** paraformaldehyde and subsequently incubated with primary antibodies resuspended in the FoxP3/transcription factor buffer staining set (eBioscience). For IL-12 and TNF-α staining, cells were incubated for 6 hrs with Brefeldin-A only (eBioscience). Intracellular staining experiments used 10^6^ cells. Antibodies used for intracellular staining included anti-IFN-γ PE-Cy7, anti-TNF-α PE-Cy7 (Biolegend); anti-IL-12 PE, anti-TNF-α APC (BD Biosciences), and anti-*Toxoplasma* p30 (Argene, Shirley, NY). Cell fluorescence was measured using a FACS Canto (BD Biosciences). Data was analyzed using FlowJo software (FlowJo, Ashland, OR).

### Quantitative PCR for parasite burden

DNA was isolated from whole intestinal tissue using a Tissue DNA kit following manufacturer's instructions (Omega Biotek, Norcross, GA). DNA was amplified by quantitative PCR as described previously using primers against the *T. gondii* B1 gene and the host argininosuccinate lyase (ASL) gene [61]. Ten-fold serial dilutions of genome copy standard curves were created using known quantities of host (splenocytes) and parasite (tachyzoites) cells based on DNA quantity, Avogadro's number, and genome size. To quantify parasite burden, the generated values for host and parasite genome copies from the DNA preparations were expressed as a ratio of parasite (B1) to host (ASL) genomes.

### Adoptive transfer

Splenocytes from naïve mice were harvested and subjected to CD4 positive selection by magnetic bead sorting following manufacturer's instructions (Stem Cell Technologies, Vancouver, British Columbia). Cells were purified to ∼90–95% purity and were transferred by intravenous retro-orbital injection into *Cxcr3^−/−^* recipients at 5×10^6^ CD4^+^ cells per mouse. Twenty-four hours post transfer, mice were challenged with 30 cysts of the *T. gondii* ME49 strain. In some experiments mice were left to assess survival following cell transfer. In other experiments intestinal tissue was collected at day 9 post-infection for intracellular cytokine analysis.

### Pathology scoring

Swiss rolls of the intestines were histopathologically scored by an investigator that was double-blinded to sample identity. Intestines were scored on an ascending 0–4 scale as previously described [50], [62]. Briefly, scores of 0 were normal, scores of 1 indicated mild focal lesions, scores of 2 indicated moderate focal lesions, scores of 3 indicated moderate multifocal lesions, and scores of 4 indicated severe multifocal lesions. Histopathological features scored included: inflammation of the intestinal submucosa (lamina propria), inflammation extending throughout all histological layers of the intestine (transmural inflammation), sloughing of intestinal epithelium, intestinal villus fusion and blunting, and necrosis of villi. Sections of complete small intestines from 13 KO and 14 WT infected mice were scored. The data are plotted as mean scores of individual mice.

### Statistics

Differences between groups were analyzed using student's *t*-test. Differences between 3 or more groups were analyzed using one-way Anova with Newman-Keuls post-test. P-values less than 0.05 are considered significant and are designated by * p<0.05, ** p<0.01, or *** p<0.001. Pathology scores were analyzed using the Mann-Whitney *t*-test.

## Supporting Information

Figure S1 **CXCR3-GFP expression on NK cells decreases during infection.** Lamina propria and mesenteric lymph node (MLN) leukocytes were isolated from noninfected (NI) and Day 7-infected (INF) CIBER reporter mice, and CXCR3-GFP expression was measured on NK1.1^+^ cells. Numbers in each panel indicate the percent of NK1.1^+^ cells falling within the CXCR3-eGFP positive quadrant.
(TIF)

Figure S2 **CXCR3-GFP^+^ and CXCR3-GFP^−^ fractions of CD4^+^ T cells differentially express activation markers.** Splenocytes (n = 4), MLN (n = 5), and Peyer's patch (n = 2) cells were isolated from Day 11-infected CIBER reporter mice and stained for surface markers CD27 as well as CD4 (A and B) and CD8 (C and D). Representative mice are shown in A and C. Bar graphs (B and D) represent averages of multiple mice, and significance is represented by * p<0.05, and *** p<0.001.
(TIF)

Figure S3 ***Cxcr3^−/−^*** **mice are more susceptible to oral infection with** ***Toxoplasma*** **.** (A) WT and KO mice (n = 5 per group) were infected by i.p. injection of 30 ME49 cysts, and survival was monitored. (B) Small intestines of naïve WT and KO mice (n = 3 per strain) were harvested, fixed in formaldehyde, embedded in paraffin, and sections were stained with H&E. (C) Frozen sections of Day 10 WT and *Cxcr3^−/−^* intestines were stained for Muc1 followed by anti-rabbit Alexa-488 (red). Sections were counter-stained with DAPI (blue). (D) Lamina propria leukocytes from Day 9-orally infected WT and *Cxcr3^−/−^* mice were stained for neutrophil markers Ly6C/G (Gr-1) and Ly6G (1A8). (E) Neutrophil levels were assessed in individual mice (n = 5 per group). The graph shows mean +/− SEM (** p<0.01).
(TIF)

Figure S4 **Cytokine responses in WT and KO mice.** Splenocytes (A, C and E) and MLN (B, D and F) were harvested from Day 11-infected WT and *Cxcr3^−/−^* mice and cultured in the presence of soluble tachyzoite antigen (STAg) for 72 hr. Supernatants were collected, and IFN-γ (A and B), TNF-α (C and D), and IL-10 (E and F) were measured by ELISA.
(TIF)

Figure S5 **T cell and NK cell production of IFN-γ in the presence and absence of CXCR3.** Lamina propria leukocytes were isolated from WT (A) and *Cxcr3^−/−^* (B) mice 6 days post-infection, cultured for 6 hr in the presence of PMA, ionomycin, and Brefeldin-A, and stained for CD8 and IFN-γ. (C) Means and standard errors of individual mice. Lamina propria leukocytes were isolated from WT (D) and *Cxcr3^−/−^* (E) mice, cultured in the presence of PMA, ionomycin, and Brefeldin-A and stained for NK1.1 and IFN-γ. (F) Mean and standard error of individual mice (WT: n = 9; KO: n = 6). Splenocytes were harvested from WT (G) and *Cxcr3^−/−^* (H) Day 7-infected mice, cultured in the presence of PMA, ionomycin, and Brefeldin-A, and stained for CD4 and IFN-γ. (I) Mean and standard error of individual mice (WT: n = 2; KO: n = 2).
(TIF)

Figure S6 **Lamina propria neutrophils secrete elevated TNF-α in the absence of CXCR3.** Lamina propria leukocytes were harvested from WT and *Cxcr3^−/−^* mice and cultured in the presence of Brefeldin-A for 6 hr. Cells were then stained for neutrophil markers CD11b, Ly6C/G (Gr-1), and Ly6G (1A8), fixed, permeabilized, and intracellularly stained for TNF-α. Cytokine production was assessed by flow cytometry (A). Shown are the mean +/− SEM of individual mice (B) (n = 5 per group, ** p<0.01).
(TIF)

Figure S7 **Adoptive transfer of CXCR3^+^CD4^+^ T cells into** ***Cxcr3^−/−^*** **recipients protects against oral** ***Toxoplasma*** **infection.** (A) Splenic CD4^+^ T cells were isolated from naive *Cxcr3^−/−^* mice, and 5×10^6^ cells were adoptively transferred intravenously into *Cxcr3^−/−^* recipients. Mice were orally challenged 24 hr later with 30 ME49 cysts and assessed for survival (n = 5 mice per group). (B) Lamina propria leukocytes were harvested from infected WT and *Cxcr3^−/−^* mice and cultured in the presence of Brefeldin-A for 6 hr. Cells were surface stained for CD11b, Ly6C/G (Gr-1), and Ly6G to identify inflammatory monocytes. Cells were then fixed, permeabilized, and intracellularly stained for IL-12 and TNF-α. Cytokine production by inflammatory monocytes was analyzed by flow cytometry. Shown are representative FACS plots of individual mice. (C) Lamina propria leukocytes were surface stained for CD11b, Ly6C/G (Gr-1), and Ly6G (1A8) to identify neutrophils. (D) Cells were then fixed, permeabilized, and intracellularly stained for TNF-α. Cytokine production by neutrophils was analyzed by flow cytometry. Shown are the mean +/− SEM of individual mice (n = 5 mice per group; * p<0.05, ** p<0.01).
(TIF)
