# Supplementary figures and images for: CXCR3-Dependent CD4+ T Cells Are Required to Activate Inflammatory Monocytes for Defense against Intestinal Infection
Source: PLoS Pathog. 2013 Oct 10;9(10):e1003706. doi: 10.1371/journal.ppat.1003706 (PMC3795032; doi:10.1371/journal.ppat.1003706)

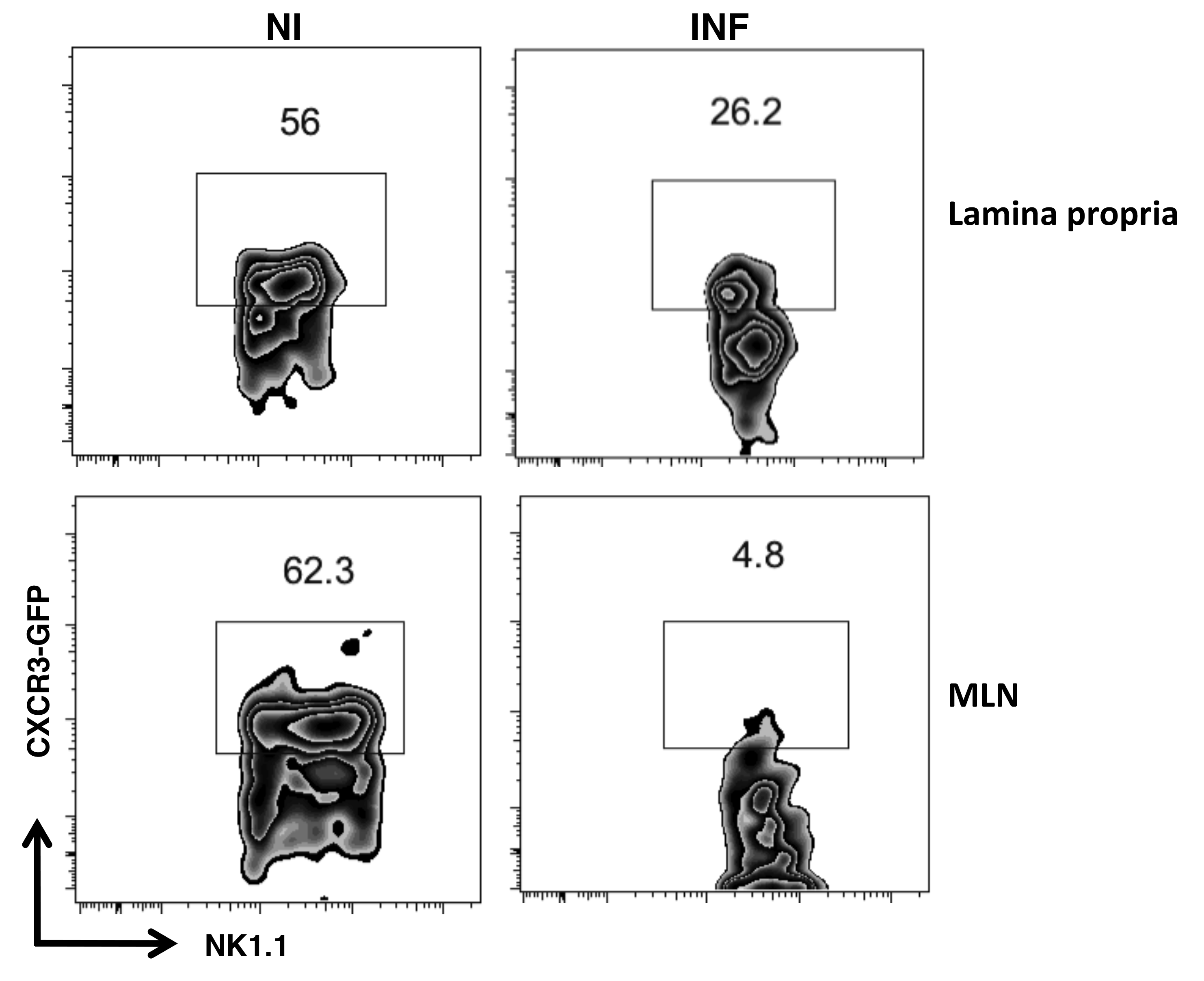

Supplement: Figure S1 — CXCR3-GFP expression on NK cells decreases during infection. Lamina propria and mesenteric lymph node (MLN) leukocytes were isolated from noninfected (NI) and Day 7-infected (INF) CIBER reporter mice, and CXCR3-GFP expression was measured on NK1.1+ cells. Numbers in each panel indicate the percent of NK1.1+ cells falling within the CXCR3-eGFP positive quadrant. (TIF) [file ppat.1003706.s001.tif]

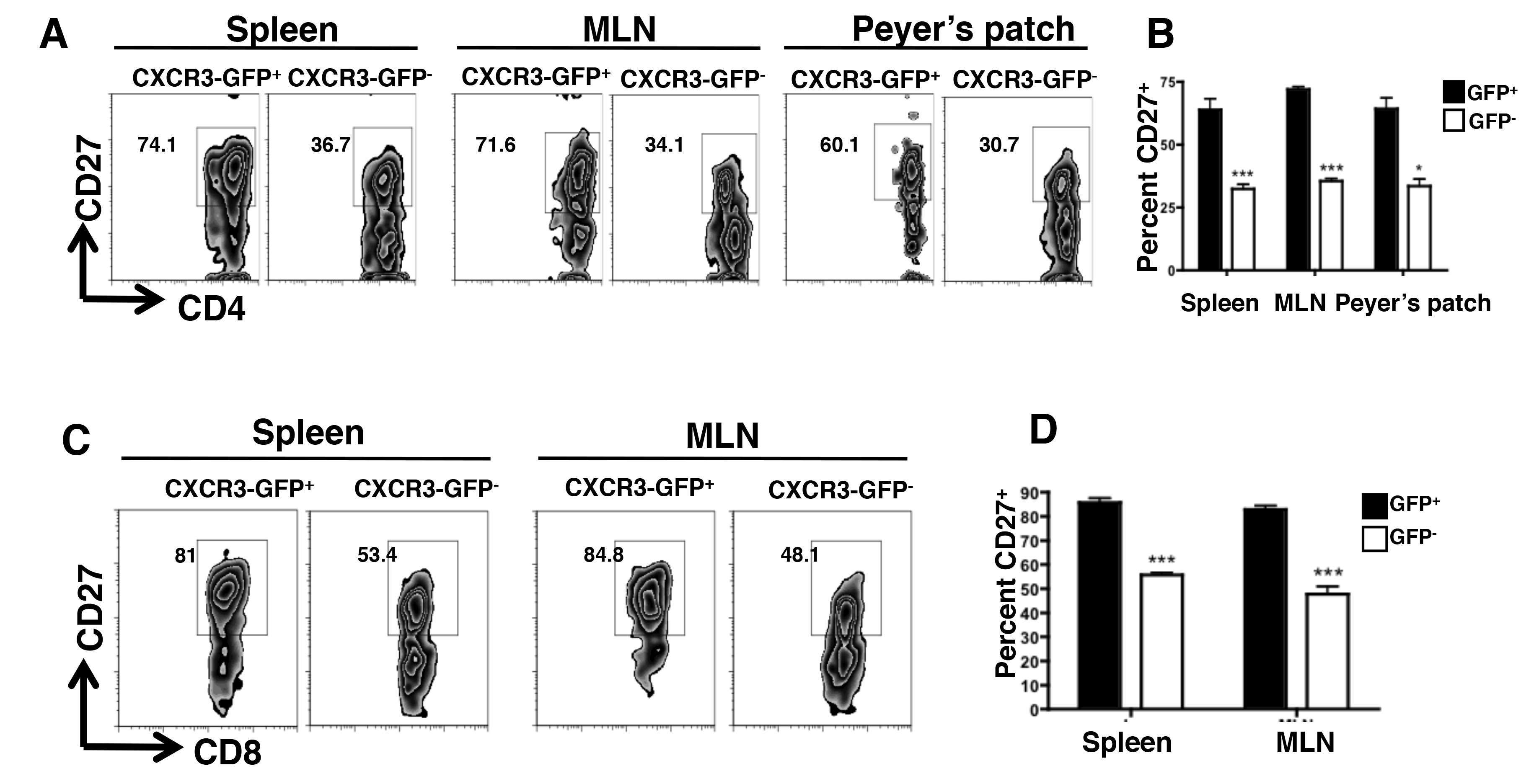

Supplement: Figure S2 — CXCR3-GFP+ and CXCR3-GFP− fractions of CD4+ T cells differentially express activation markers. Splenocytes (n = 4), MLN (n = 5), and Peyer's patch (n = 2) cells were isolated from Day 11-infected CIBER reporter mice and stained for surface markers CD27 as well as CD4 (A and B) and CD8 (C and D). Representative mice are shown in A and C. Bar graphs (B and D) represent averages of multiple mice, and significance is represented by * p<0.05, and *** p<0.001. (TIF) [file ppat.1003706.s002.tif]

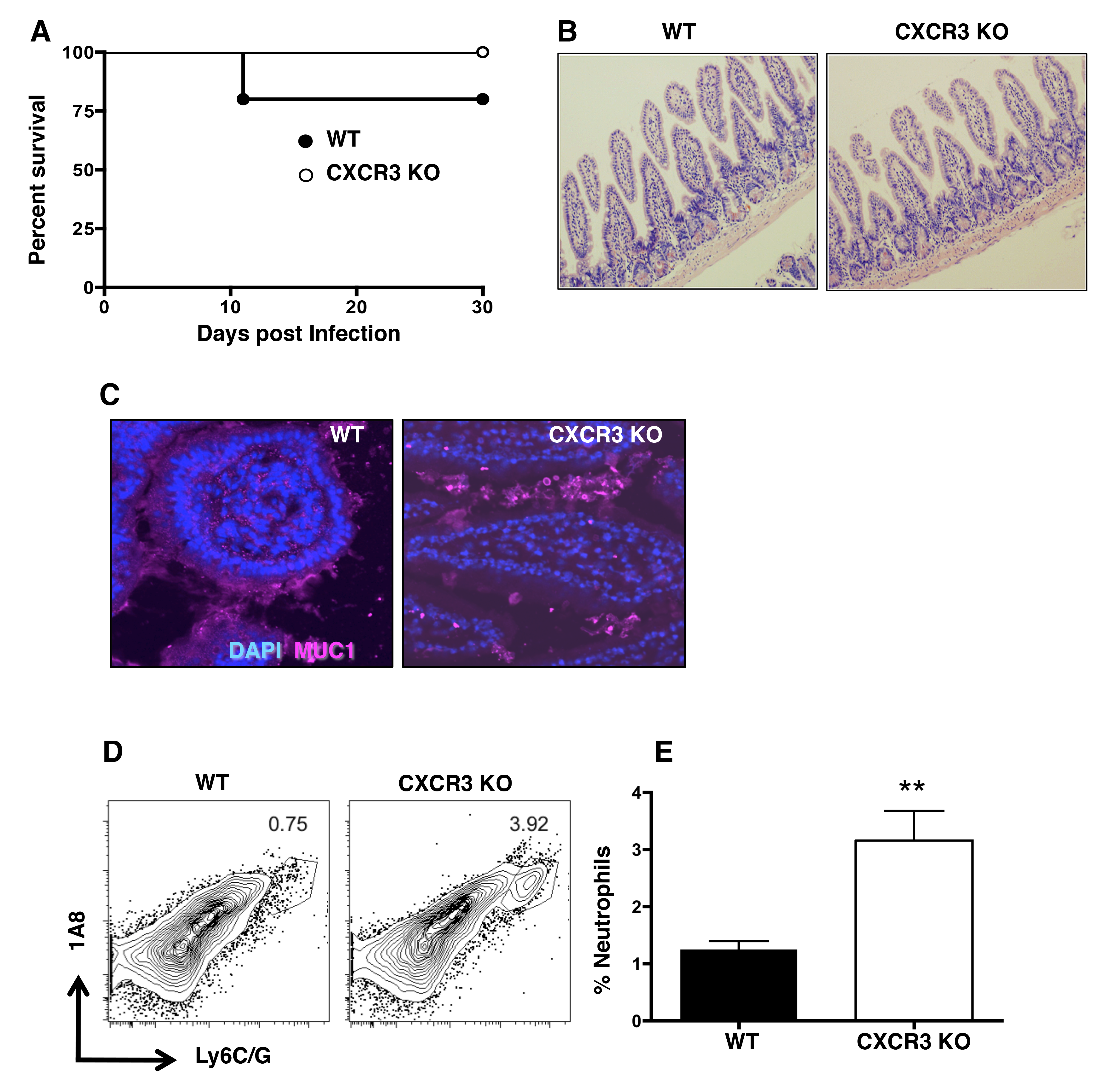

Supplement: Figure S3 — Cxcr3−/− mice are more susceptible to oral infection with Toxoplasma . (A) WT and KO mice (n = 5 per group) were infected by i.p. injection of 30 ME49 cysts, and survival was monitored. (B) Small intestines of naïve WT and KO mice (n = 3 per strain) were harvested, fixed in formaldehyde, embedded in paraffin, and sections were stained with H&E. (C) Frozen sections of Day 10 WT and Cxcr3−/− intestines were stained for Muc1 followed by anti-rabbit Alexa-488 (red). Sections were counter-stained with DAPI (blue). (D) Lamina propria leukocytes from Day 9-orally infected WT and Cxcr3−/− mice were stained for neutrophil markers Ly6C/G (Gr-1) and Ly6G (1A8). (E) Neutrophil levels were assessed in individual mice (n = 5 per group). The graph shows mean +/− SEM (** p<0.01). (TIF) [file ppat.1003706.s003.tif]

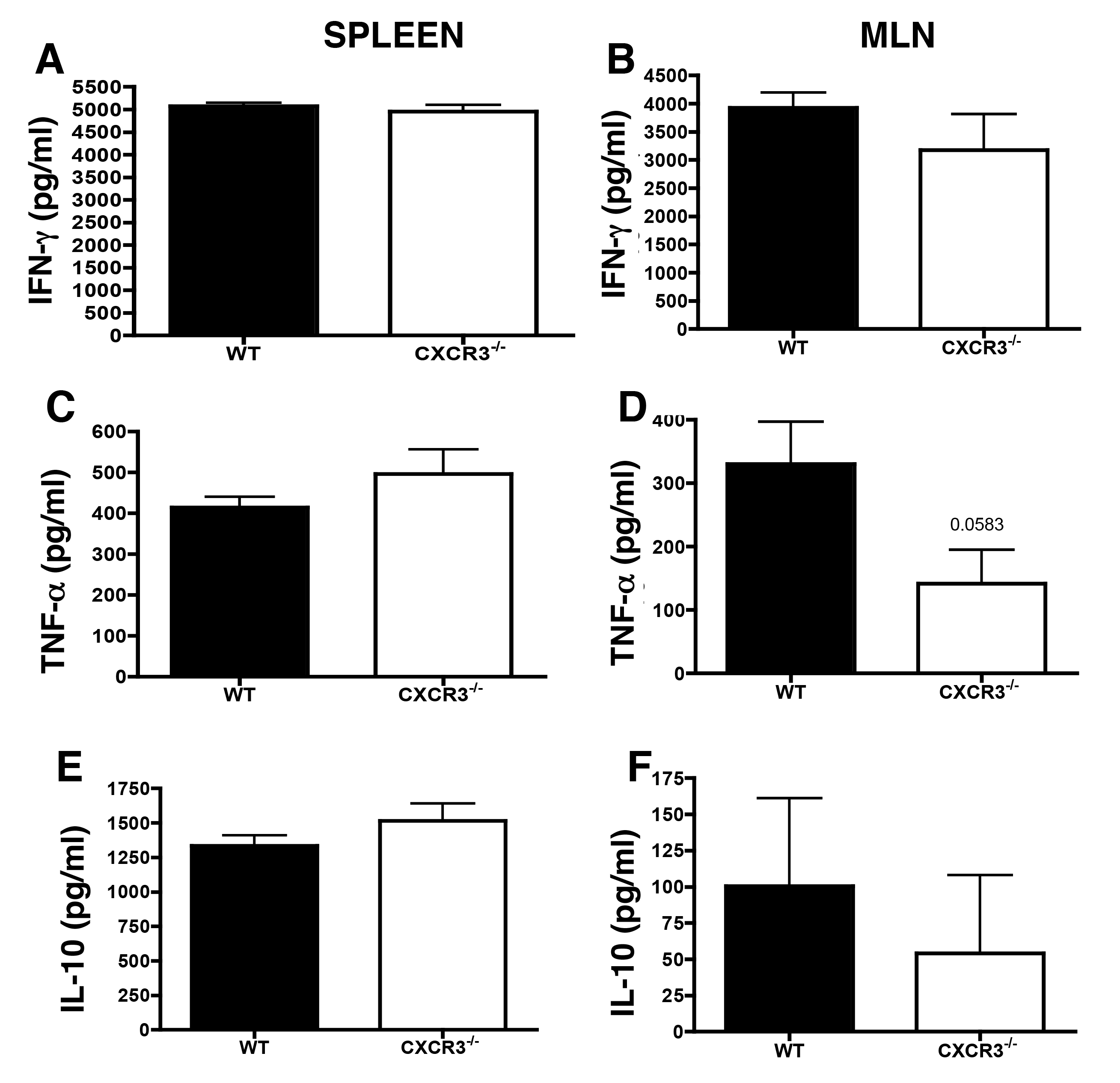

Supplement: Figure S4 — Cytokine responses in WT and KO mice. Splenocytes (A, C and E) and MLN (B, D and F) were harvested from Day 11-infected WT and Cxcr3−/− mice and cultured in the presence of soluble tachyzoite antigen (STAg) for 72 hr. Supernatants were collected, and IFN-γ (A and B), TNF-α (C and D), and IL-10 (E and F) were measured by ELISA. (TIF) [file ppat.1003706.s004.tif]

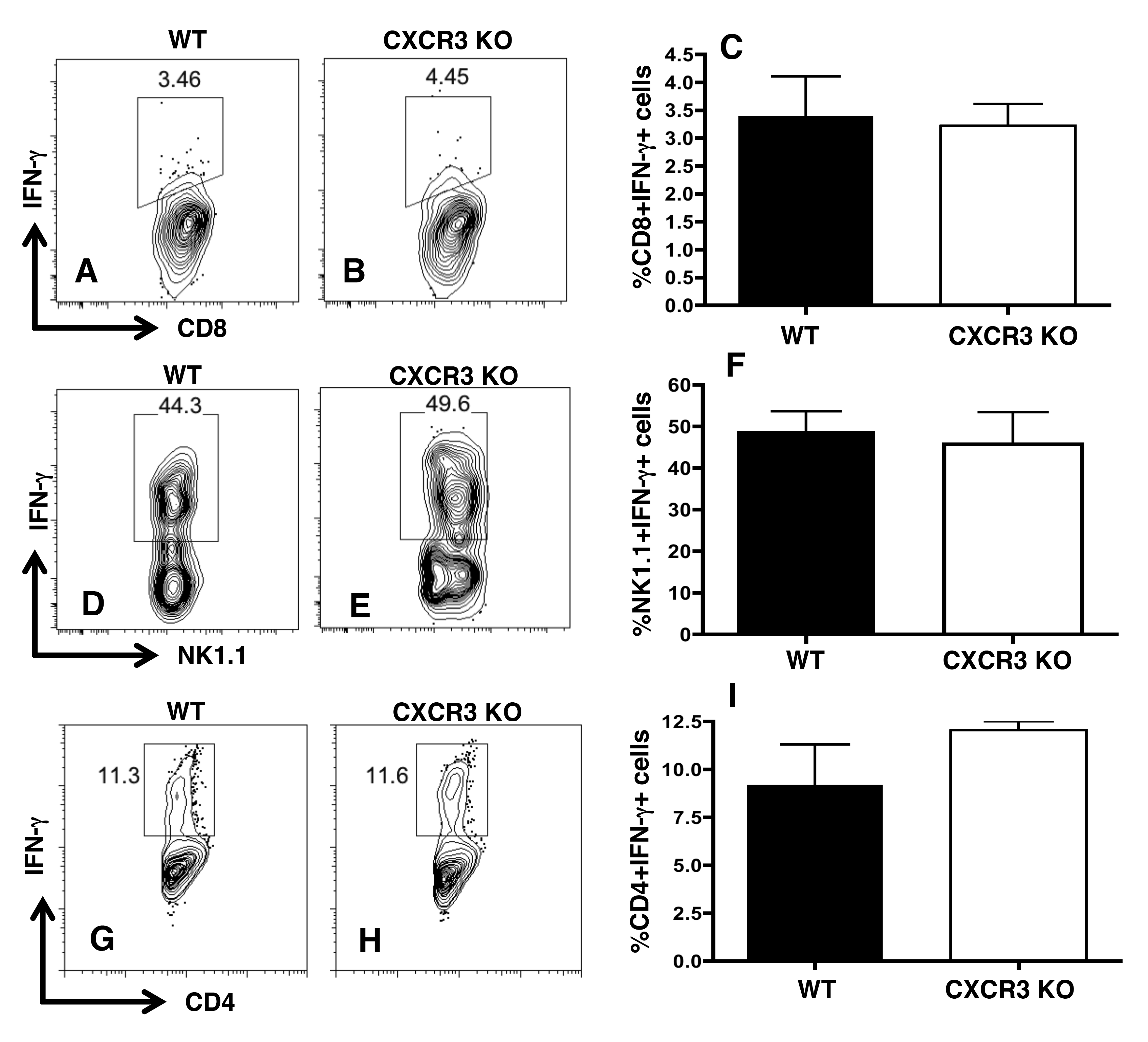

Supplement: Figure S5 — T cell and NK cell production of IFN-γ in the presence and absence of CXCR3. Lamina propria leukocytes were isolated from WT (A) and Cxcr3−/− (B) mice 6 days post-infection, cultured for 6 hr in the presence of PMA, ionomycin, and Brefeldin-A, and stained for CD8 and IFN-γ. (C) Means and standard errors of individual mice. Lamina propria leukocytes were isolated from WT (D) and Cxcr3−/− (E) mice, cultured in the presence of PMA, ionomycin, and Brefeldin-A and stained for NK1.1 and IFN-γ. (F) Mean and standard error of individual mice (WT: n = 9; KO: n = 6). Splenocytes were harvested from WT (G) and Cxcr3−/− (H) Day 7-infected mice, cultured in the presence of PMA, ionomycin, and Brefeldin-A, and stained for CD4 and IFN-γ. (I) Mean and standard error of individual mice (WT: n = 2; KO: n = 2). (TIF) [file ppat.1003706.s005.tif]

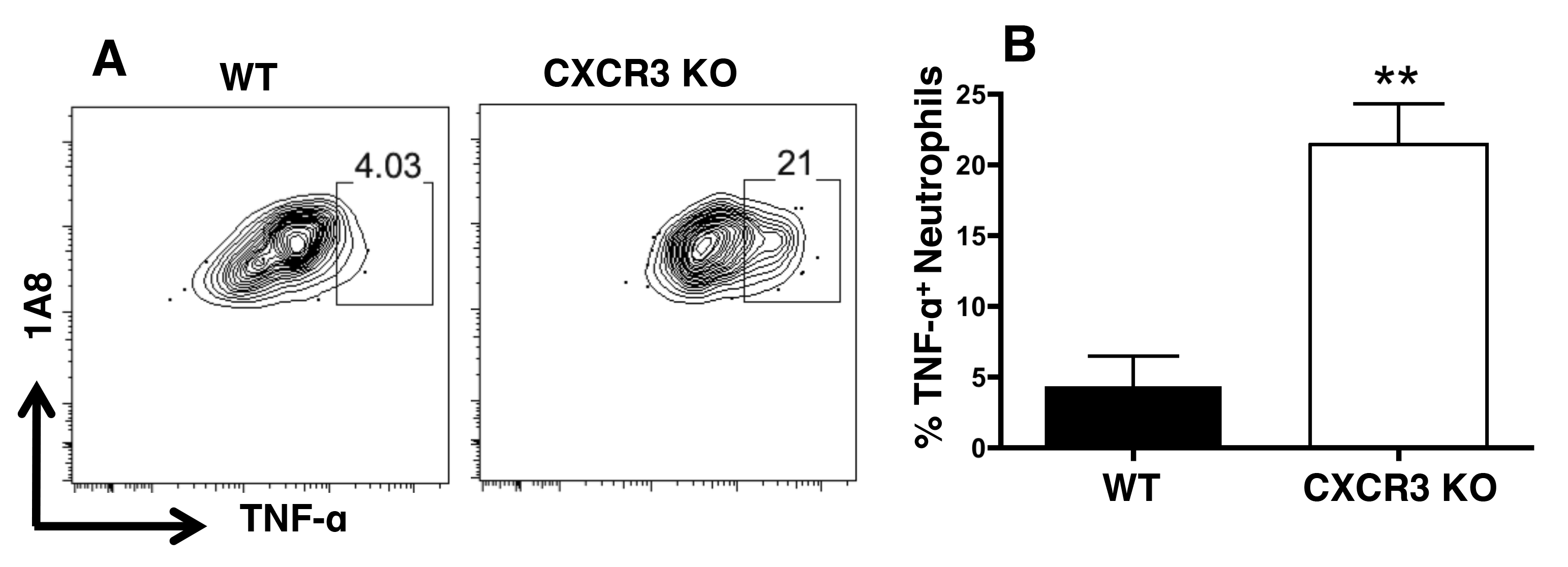

Supplement: Figure S6 — Lamina propria neutrophils secrete elevated TNF-α in the absence of CXCR3. Lamina propria leukocytes were harvested from WT and Cxcr3−/− mice and cultured in the presence of Brefeldin-A for 6 hr. Cells were then stained for neutrophil markers CD11b, Ly6C/G (Gr-1), and Ly6G (1A8), fixed, permeabilized, and intracellularly stained for TNF-α. Cytokine production was assessed by flow cytometry (A). Shown are the mean +/− SEM of individual mice (B) (n = 5 per group, ** p<0.01). (TIF) [file ppat.1003706.s006.tif]

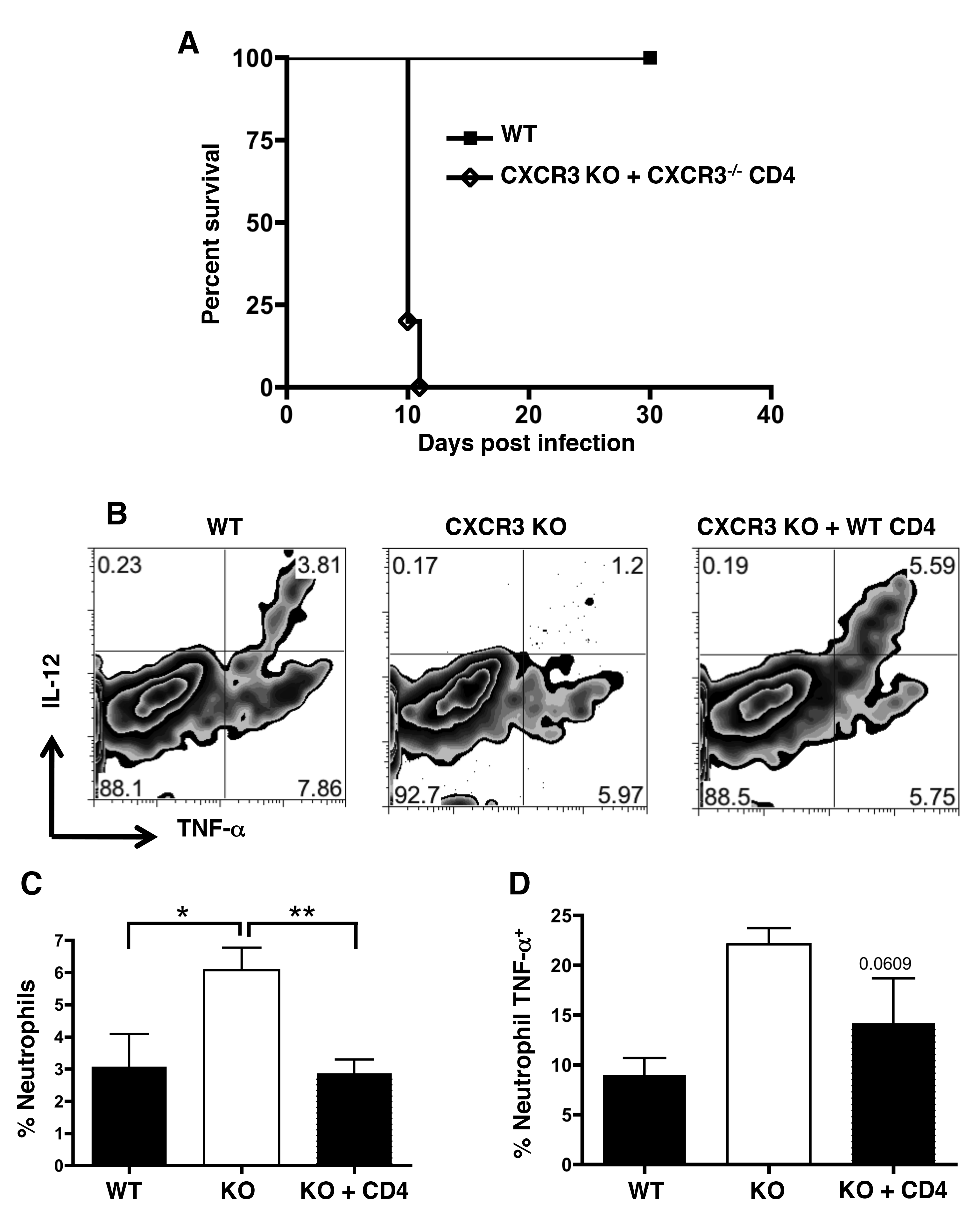

Supplement: Figure S7 — Adoptive transfer of CXCR3+CD4+ T cells into Cxcr3−/− recipients protects against oral Toxoplasma infection. (A) Splenic CD4+ T cells were isolated from naive Cxcr3−/− mice, and 5×10^6^ cells were adoptively transferred intravenously into Cxcr3−/− recipients. Mice were orally challenged 24 hr later with 30 ME49 cysts and assessed for survival (n = 5 mice per group). (B) Lamina propria leukocytes were harvested from infected WT and Cxcr3−/− mice and cultured in the presence of Brefeldin-A for 6 hr. Cells were surface stained for CD11b, Ly6C/G (Gr-1), and Ly6G to identify inflammatory monocytes. Cells were then fixed, permeabilized, and intracellularly stained for IL-12 and TNF-α. Cytokine production by inflammatory monocytes was analyzed by flow cytometry. Shown are representative FACS plots of individual mice. (C) Lamina propria leukocytes were surface stained for CD11b, Ly6C/G (Gr-1), and Ly6G (1A8) to identify neutrophils. (D) Cells were then fixed, permeabilized, and intracellularly stained for TNF-α. Cytokine production by neutrophils was analyzed by flow cytometry. Shown are the mean +/− SEM of individual mice (n = 5 mice per group; * p<0.05, ** p<0.01). (TIF) [file ppat.1003706.s007.tif]
